# Gene silencing by EZH2 suppresses TGF-β activity within the decidua to avert pregnancy-adverse wound healing at the maternal-fetal interface

**DOI:** 10.1016/j.celrep.2022.110329

**Published:** 2022-02-01

**Authors:** Ivan Osokine, Johan Siewiera, Damon Rideaux, Stephany Ma, Tatsuya Tsukui, Adrian Erlebacher

**Affiliations:** 1 Department of Laboratory Medicine, University of California San Francisco, 513 Parnassus Avenue Medical Sciences, S-1057B, San Francisco, CA 94143-0451, USA; 2 Lung Biology Center, Department of Medicine, University of California San Francisco, San Francisco, CA 94143, USA; 3 Center for Reproductive Sciences, University of California San Francisco, San Francisco, CA 94143, USA; 4 Biomedical Sciences Program, University of California San Francisco, San Francisco, CA 94143, USA; 5 Bakar ImmunoX Initiative, University of California San Francisco, San Francisco, CA 94143, USA; 6 Lead contact

## Abstract

A little-appreciated feature of early pregnancy is that embryo implantation and placental outgrowth do not evoke wound-healing responses in the decidua, the specialized endometrial tissue that surrounds the conceptus. Here, we provide evidence that this phenomenon is partly due to an active program of gene silencing mediated by EZH2, a histone methyltransferase that generates repressive histone 3 lysine 27 trimethyl (H3K27me3) histone marks. We find that pregnancies in mice with EZH2-deficient decidual stromal cells frequently fail by mid-gestation, with the decidua showing ectopic myofibroblast formation, peri-embryonic collagen deposition, and gene expression profiles associated with transforming growth factor β (TGF-β)-driven fibroblast activation and fibrogenic extracellular matrix (ECM) remodeling. Analogous responses are observed when the mutant decidua is surgically wounded, while blockade of TGF-β receptor signaling inhibits the defects and improves reproductive outcomes. Together, these results highlight a critical feature of reproductive success and have implications for the context-specific control of TGF-β-mediated wound-healing responses elsewhere in the body.

## In brief

Osokine et al. demonstrate that the epigenetic regulator EZH2 renders decidual stromal cells resistant to wound-healing signals mediated by TGF-β. Loss of EZH2 from the decidua results in fibroblast activation, induction of TGF-β target genes, and pregnancy failure. *In vivo* TGF-βRI inhibition partially rescues these effects.

## Graphical abstract

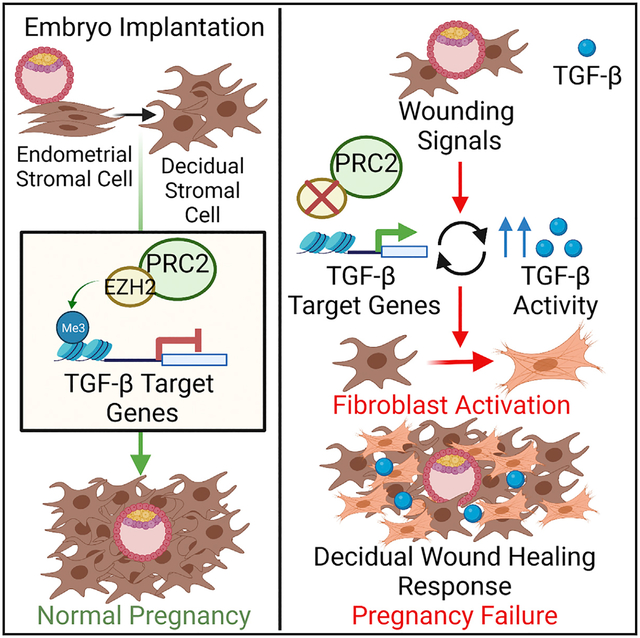

## INTRODUCTION

The dynamic deposition and erasure of histone 3 lysine 27 trimethyl (H3K27me3) marks on genetic loci has emerged as a crucial determinant of cell differentiation and function (for a review, see Margueron and Reinberg, 2011). Generated by polycomb repressive complex 2 (PRC2) through its mutually exclusive catalytic subunits enhancer of zeste homolog 1 (EZH1) and EZH2, H3K27me3 marks induce transcriptional silencing, whereas H3K27 demethylation promotes gene expression. Although H3K27me3 dynamics are frequently studied in the context of embryonic development, they also contribute to processes such as host defense and wound healing in adult organisms (Busslinger and Tarakhovsky, 2014; Lewis et al., 2014).

Previously, we uncovered evidence suggesting a role for EZH2 in decidualization, the process by which the endometrial lining of the uterus transforms into the decidua during pregnancy (Nancy et al., 2012, 2018). The decidua serves as the maternal component of the maternal-fetal interface, providing signals that promote embryo implantation and invasion, modulating the local immune environment, and providing structural support to the placenta (for reviews, see Cha et al., 2012 and Wagner et al., 2014). It is primarily comprised of a dense network of decidual stromal cells (DSCs) that arise from the endometrial stromal cells (ESCs) of the endometrium. In mice, a species in which decidualization is triggered locally around each embryo, we found that the transformation of ESCs into DSCs is associated with a dramatic induction of EZH2 expression, concomitant with cells’ *de novo* accumulation of H3K27me3 marks on ~800 protein-coding genes and the transcriptional silencing of many of them (Nancy et al., 2018). Although uterine EZH2 has been found to regulate the epithelium of the undecidualized endometrium (Fang et al., 2020; Nanjappa et al., 2019), these observations suggested that EZH2 also serves a unique function within the decidua. Indeed, given the set of affected genes, the silencing program potentially explained our prior observation that the decidua is unable to recruit activated T cells from the bloodstream or to homeostatically expand its pool of macrophages (Nancy et al., 2012, 2018; Tagliani et al., 2011).

Notably, many of the silenced genes also had known roles in the formation and function of myofibroblasts, a contractile fibroblast subtype generated during wound healing from activated fibroblasts (for a review, see Hinz et al., 2012). This observation suggested that H3K27me3 deposition in DSCs might also suppress certain features of wound healing, raising the question of why such a regulatory mechanism might be necessary for pregnancy success. One possibility is that the disruption of uterine tissue caused by embryo implantation and subsequent placental outgrowth may itself serve as a potential wounding stimulus. Indeed, it is remarkable that acute inflammation, granulation tissue formation, myofibroblast formation, and fibrosis—all key features of wound healing (Bonnans et al., 2014)—are not apparent at the maternal-fetal interface. However, this arrangement makes physiological sense since healing of the implantation site’s uterine “wound” could interfere with embryo development and/or result in termination of the pregnancy.

Here, we directly assess the possibility that EZH2 attenuates wound healing within the decidua by employing a conditional EZH2 knockout model (*Ezh2* cKO) in which the *Ezh2* gene is deleted within DSCs in mice. Such targeted EZH2 loss led to mid-gestation pregnancy failure associated with an intra-DSC reaction similar to that seen following wounding. This reaction was, in turn, the result of derepressed activity of transforming growth factor β (TGF-β), the signature cytokine that drives myofibroblast formation (Hinz et al., 2012). Notably, TGF-β receptor signaling within DSC is known to be necessary for decidualization (Fang et al., 2020; Ni and Li, 2017; Peng et al., 2015), suggesting that PRC2-mediated gene silencing within DSCs functions to selectively attenuate only pregnancy-adverse features of the overall potential intra-decidual TGF-β response.

## RESULTS

### *Pgr*-cre *Ezh2*^*f/f*^ mice show mid-gestation pregnancy failure associated with abnormal decidualization

To generate *Ezh2* cKO (i.e., *Pgr*^*cre/+*^ *Ezh2*^*fl/fl*^) mice, we intercrossed C57BL/6-background *Ezh2*^*fl/fl*^ mice (Su et al., 2003) with C57BL/6 mice bearing a *Pgr*-cre driver (Soyal et al., 2005), which targets gene deletion to progesterone receptor (PR)-expressing reproductive tissues. Age-matched *Pgr*^+/+^ *Ezh2*^*fl/fl*^ littermates were used as controls. Importantly, a lineage-tracing analysis of uterine cells of early pregnancy using a double-fluorescent cre reporter mouse (Muzumdar et al., 2007) revealed *Pgr*-cre-mediated recombination in DSCs and epithelial cells, as expected, but not in decidual endothelial cells nor leukocytes (Figure S1), with the absence of recombination in decidual leukocytes consistent with the lack of PR expression by immune cells systemically (Heng et al., 2008).

**Figure 1.**
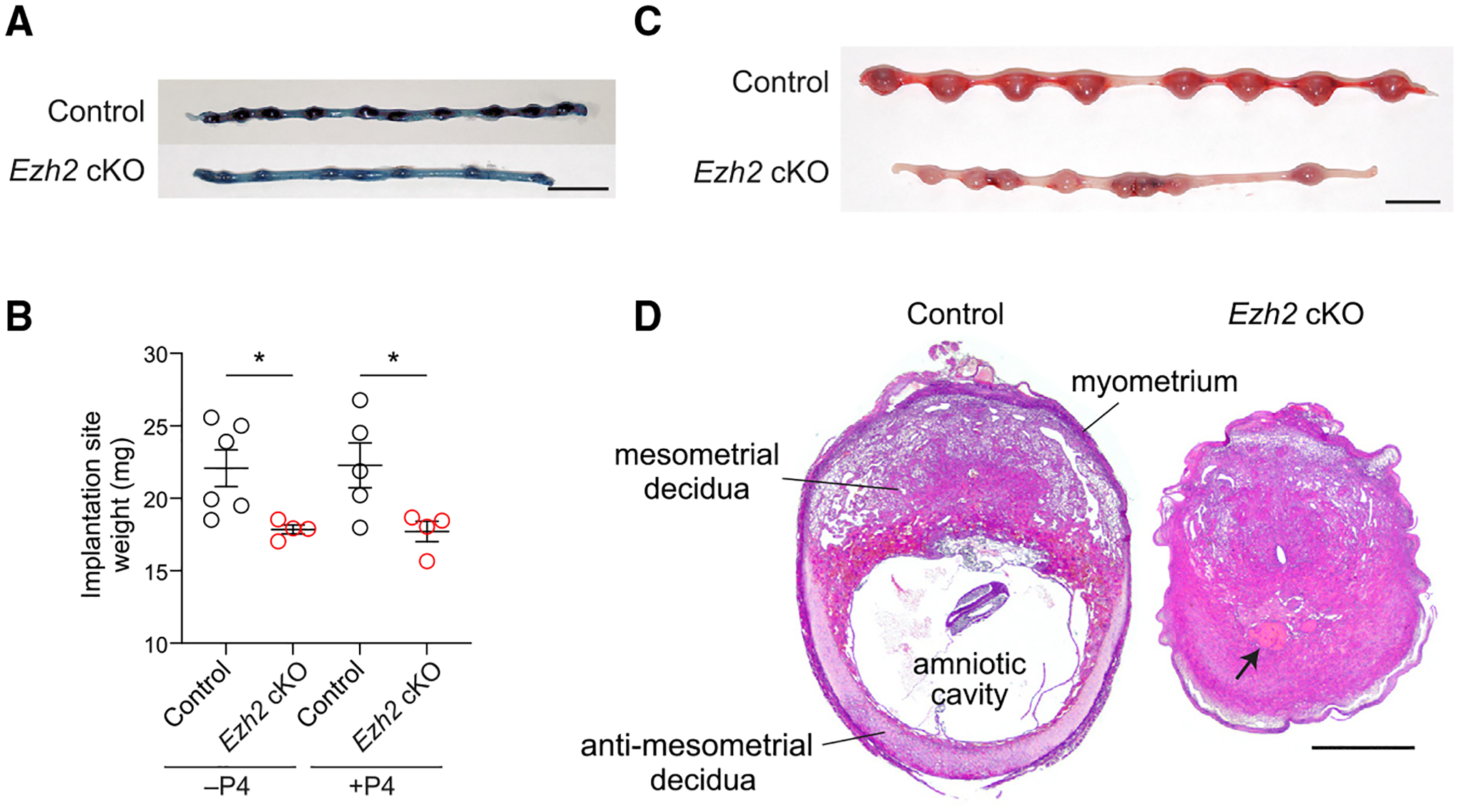
Post-implantation pregnancy failure in *Ezh2* cKO mice (A) Whole-mount photographs of representative uteri on E5.5 (n = 6 mice/group). Chicago sky blue dye was administered immediately prior to sacrifice to highlight implantation sites (dark blue). Scale bar, 1 cm. (B) Implantation site weights on E7.5. As indicated, mice were otherwise left untreated (−P4) or were injected daily with 2 mg P4 starting on E5.5 (+P4). Data show mean ± SEM of the average implantation site weight for each mouse. n = 4–6 mice/group; *p < 0.05, Student’s t test. (C and D) Whole-mount photographs (C) and H&E stains (D) of representative uteri and implantation sites on E9.5 from mice that were injected daily with 2 mg P4 starting on E5.5. Implantation site architecture is labeled in (D), and the arrow indicates a necrotic embryo. Embryos in other *Ezh2* cKO implantation sites frequently appeared stunted (e.g., Figures 2D and S2B) or hemorrhagic (e.g., Figure 6B). Note that the *Ezh2* cKO implantation site also lacks an amniotic cavity. n = 5 mice/group. Scale bars, 1 cm (C) and 500 μm (D).

As mentioned, two studies previously revealed that EZH2 regulates the non-pregnant uterine epithelium (Fang et al., 2019; Nanjappa et al., 2019). These studies, which also employed *Ezh2* cKO mice but on a mixed C57BL/6; 129Sv background, noted the onset of uterine epithelial hyperplasia starting at ~1 month of age. They also noted a progressive loss of fertility starting at ~5 months of age associated with decreased live litter numbers and ~50% smaller litter sizes at birth yet normal implantation site numbers in early gestation. Our line of *Ezh2* cKO mice also showed uterine hyperplasia (data not shown) as well as a fertility impairment associated with decreased live litter numbers, but this impairment was apparent as early as 8–12 weeks of age (Figure S2A), the age of all the mice used for the rest this study. For those mice that were pregnant, litter sizes were normal both in early gestation (i.e., embryonic day 7.5 [E7.5]; 7.8 versus 7.3 pups per litter, respectively, p = 0.45, Student’s t test, n = 12 mice/group) as well as at birth (Figure S2A). While confirming the overall subfertility of *Ezh2* cKO mice, these observations thus revealed some subtle and potentially strain-background-dependent phenotypic variations. We chose not to pursue these further. Rather, we focused on an additional observation, namely that implantation sites in *Ezh2* cKO mice, despite appearing normal at E5.5 (Figure 1A), were reduced in size by E7.5 (Figure 1B, left). This observation raised the possibility that the subfertility of C57BL/6 background *Ezh2* cKO mice was due, at least in part, to defective decidualization and early pregnancy failure.

Accordingly, we gave *Ezh2* cKO mice daily progesterone (P4) injections starting at E5.5 until 24 h before sacrifice to test whether their small implantation sites were due to decidua-intrinsic defects as opposed to endocrine disruption secondary to *Pgr*-cre-mediated recombination in the ovaries or pituitary gland. P4 supplementation, while by itself not expected to impair decidualization (Erlebacher et al., 2004), also ensured technical consistency with the experiments described below that employed pharmacological agents or surgical manipulations that could compromise ovarian function and thus necessitate P4 supplementation to reveal uterus-intrinsic processes. As shown in Figure 1B, right, implantation site weights in P4-treated *Ezh2* cKO mice were reduced compared with in P4-treated controls on E7.5, paralleling the reduction seen in non-P4-treated mice, and by E9.5, the implantation sites of P4-treated *Ezh2* cKO mice were markedly smaller than their control counterparts and frequently displayed abnormal or dead embryos and loss of the amnionic cavity (Figures 1C and 1D). As expected, EZH2 was strongly induced in control DSCs, confirming our prior work (Nancy et al., 2018), but was undetectable in DSCs of *Ezh2* cKO uteri (Figure S2B). Likewise, H3K27me3 levels in *Ezh2* cKO decidua were also dramatically reduced compared with those in control (Figure S2C). Together, these findings suggested a function for EZH2 in the early gestation decidua, when decidualization-specific H3K27me3 silencing marks are established in DSCs.

### *Ezh2* cKO mice display ectopic myofibroblast formation in the decidua and peri-embryonic collagen deposition

To identify possible causes of pregnancy failure in *Ezh2* cKO mice, we first evaluated leukocyte distributions in *Ezh2* cKO decidua, given that we previously linked H3K27me3-mediated gene silencing in DSCs to the aforementioned inability of the decidua to recruit activated T cells or to homeostatically expand its macrophage population (Nancy et al., 2012; Tagliani et al., 2011). Perhaps not surprisingly given that the mice were not manipulated to bear activated T cells, decidual T cell densities were not increased over control (Figure S3A). Macrophage densities also remained similar to control (Figure S3B). Consistent with prior work (Fang et al., 2019), we also found that natural killer (NK) cell numbers were reduced in *Ezh2* cKO decidua (Figure S3C), but this was unlikely a primary cause of pregnancy failure since mice lacking NK cells show normal fertility (Greenwood et al., 2000).

**Figure 2.**
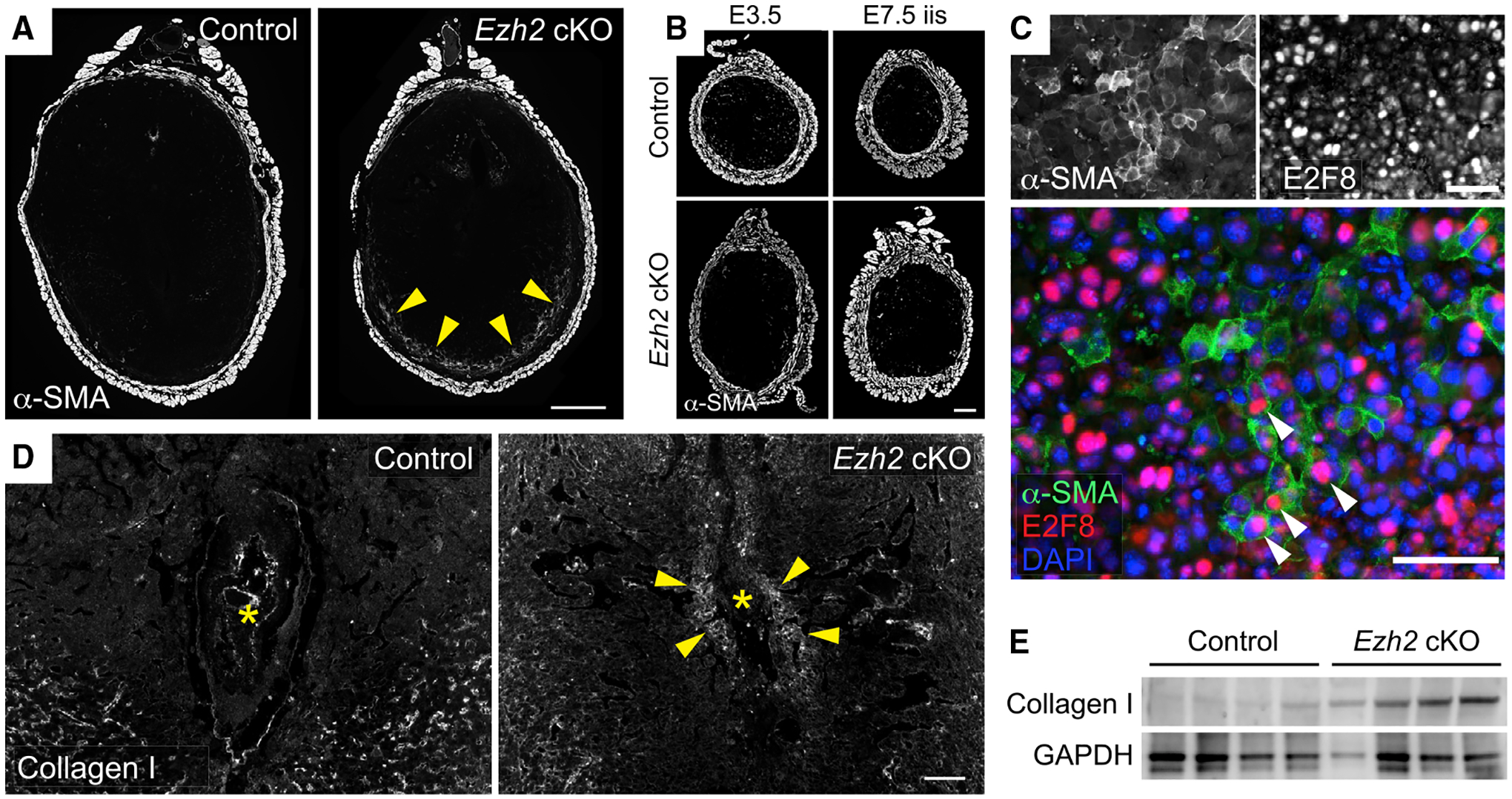
Pregnant *Ezh2* cKO mice show peri-embryonic collagen deposition and α-SMA expression by anti-mesometrial DSCs Mice sacrificed on E7.5 were injected daily with P4 starting on E5.5. (A and B) Immunofluorescence detection of α-SMA expression. Representative sections of (A) implantation sites on E7.5 (n = 6 mice/group), (B, left) undecidualized uterine segments on E3.5 (n = 4 mice/group), and (B, right) undecidualized uterine segments on E7.5 (inter-implantation sites [iiss]; n = 4 mice/group). Arrowheads indicate the ring of α-SMA^+^ cells in the anti-mesometrial decidua observed in n = 57/57 *Ezh2* cKO and 6/88 control implantation sites (p < 0.0001, Fisher’s exact test). Scale bars, 500 μm (A) and 200 μm (B). (C) Close-up image of the anti-mesometrial decidua of an *Ezh2* cKO mouse on E7.5 demonstrating nuclear E2F8 staining within α-SMA^+^ cells (white arrowheads). Scale bars, 50 μm. (D) Immunofluorescence detection of type I collagen on E7.5. Asterisks indicate embryos; arrowheads indicate peri-embryonic collagen accumulations observed in n = 5/6 *Ezh2* cKO decidua and n = 1/7 control decidua from n = 5 mice/group (p = 0.03, Fisher’s exact test). Scale bar, 100 μm. (E) Immunoblot for collagen I in E7.5 whole-tissue decidual lysates. Data show one of two independent experiments each with n = 4 mice/group.

Given these observations, we next considered that pregnancy failure might be due to an inability of DSCs to suppress myofibroblast formation, the most prominent H3K27me3-associated pathway identified by our previous chromatin immunoprecipitation sequencing (ChIP-seq)/RNA sequencing (RNA-seq) analyses of DSCs (Nancy et al., 2018). Accordingly, we immunostained pregnant uteri for α-smooth muscle actin (α-SMA), the signature myofibroblast marker and a protein encoded by one of the genes (*Acta2*) with elevated H3K27me3 levels. Strikingly, *Ezh2* cKO, but not control, implantation sites displayed a prominent band of α-SMA^+^ cells around the periphery of the anti-mesometrial decidua on E7.5 (Figure 2A, arrowheads). The appearance of these cells required decidualization, since no corresponding population was apparent in preimplantation uteri on E3.5 or in the segments of undecidualized uteri between implantation sites (inter-implantation sites [iiss]) (Figure 2B). Furthermore, the α-SMA^+^ cells expressed E2F8, a transcription factor employed by differentiated DSCs to undergo genomic endoreduplication (Qi et al., 2015) (Figure 2C), indicating that the cells were not endometrial fibroblasts that failed to decidualize.

We also noted elevated type I collagen deposition in the *Ezh2* cKO decidua (Figures 2D and S4A) as well as elevated levels of total collagen, as visualized using a fluorescent pan-collagen detection reagent (Figure S4B). Unlike the peripheral distribution of the α-SMA^+^ cells, however, these elevations were most prominent around the embryo. E7.5 *Ezh2* cKO decidua also showed increased type I collagen levels by immunoblotting (Figure 2E). By contrast, pan-collagen densities were similar in the undecidualized endometrium of control and *Ezh2* cKO mice on E3.5 and in the iiss on E7.5, except for increases around endometrial glands in *Ezh2* cKO mice (Figure S4C). Decidual collagen densities were somewhat lower in general than endometrial collagen densities, consistent with the loss of fibrillar collagen known to occur with decidualization (Diao et al., 2011). Although the above results were obtained from mice supplemented with P4, P4-un-supplemented mice showed a similar phenotype (Figures S4D–S4F). Together, the data suggested that embryo implantation and even decidualization itself generate wound-like signals that can trigger characteristic stromal cell responses (myofibroblast formation, collagen deposition) in the absence of EZH2-mediated gene silencing.

### Fibroblast activation in the *Ezh2* cKO decidua

**Figure 3.**
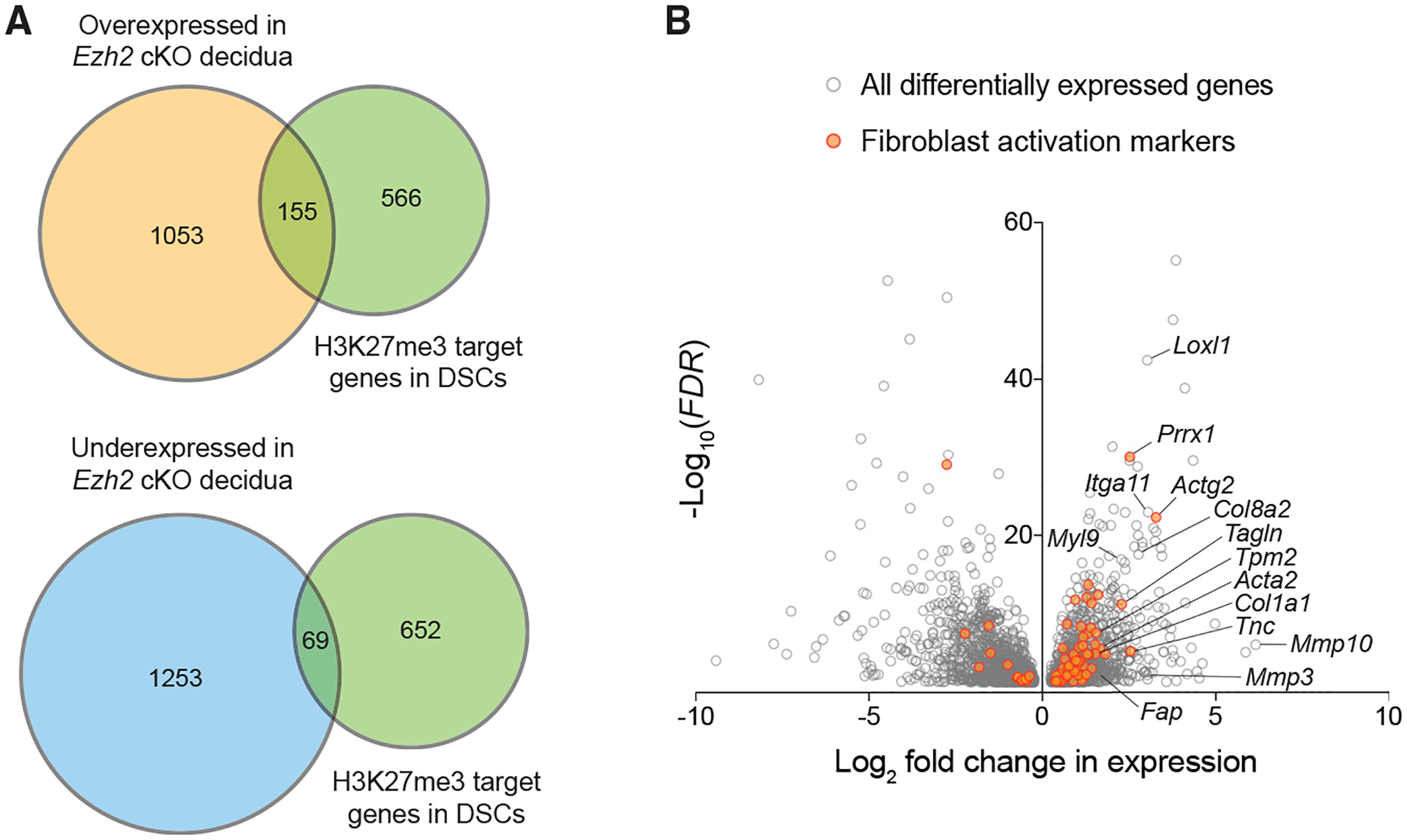
The transcriptional signature of the *Ezh2* cKO decidua is enriched for genes marked by H3K27me3 in wild-type DSCs and genes associated with fibroblast activation RNA-seq was performed on whole tissues from n = 3 *Ezh2* cKO mice and n = 4 control mice not given P4 and sacrificed on E7.5. We only considered protein-coding genes that were expressed more than 30 reads per kilobase of transcript per million mapped reads (RPKM) (14,421 total, with the RPKM for *Actb* being about 50,000). Of note, application of this expression threshold reduced the size of the H3K27me3 target-gene set in DSCs from 822 (as enumerated in our previous study without consideration of expression levels [Nancy et al., 2018]) to 721. (A) Proportional Venn diagrams demonstrating overlap of genes overexpressed or underexpressed (FDR < 0.05) in *Ezh2* cKO decidua with genes previously identified as bearing elevated H3K27me3 levels in wild-type DSCs (Nancy et al., 2018). See the main text for the hypergeometric-test-determined p values indicating whether overlapping gene sets are significantly larger or smaller than what would be expected by chance. (B) Volcano plot of expression all genes differentially expressed (FDR < 0.05) in the *Ezh2* cKO compared with in control decidua. Activated fibroblast signature genes identified by Peyser et al. (2019) and Tsukui et al. (2020) (see text) are marked in orange.

To more comprehensively determine how EZH2 deficiency in DSCs affected decidual physiology, we performed RNA-seq on whole decidual and myometrial tissues isolated from P4-untreated control and *Ezh2* cKO mice on E7.5. The myometrial tissue served as a control, given that it was our original point of comparison when we identified the set of protein-coding genes in DSCs with elevated H3K27me3 (Nancy et al., 2018). These genes will henceforth be referred to as “H3K27me3 target genes.” In *Ezh2* cKO decidua, 1,208 protein-coding genes were overexpressed and 1,322 were underexpressed (false discovery rate [FDR] < 0.05; Table S1). Of the overexpressed genes, 155 were H3K27me3 target genes (Figure 3A), which represents a 2.6-fold overrepresentation compared with chance alone (p < 1 × 10^−28^). By contrast, only 69 H3K27me3 target genes were underexpressed in *Ezh2* cKO decidua, which is not significantly different from that expected by chance (p = 0.37; Figure 3A). As expected, the impact of EZH2 deficiency on gene expression in the myometrium was much less apparent (305 overexpressed and 216 underexpressed genes; Table S2).

Importantly, the large number of differentially expressed genes in *Ezh2* cKO decidua suggested that the loss of PRC2-mediated gene silencing had broad and potentially cascading effects on decidual homeostasis. Indeed, Gene Ontogeny analysis yielded too many affected pathways to be insightful. Nonetheless, signatures of activated fibroblasts and myofibroblasts were evident among the overexpressed genes, with increased expression of both classical and emerging markers of these cell types (e.g., *Itga11*, *Tagln*, *Tpm2*, *Myl9*, *Prrx1*, *Fap*, as well as *Acta2*, as expected) (Augsten, 2014; Chang et al., 2004; Du et al., 2017; Guerrero-Juarez et al., 2019; Hinz et al., 2012; Leavitt et al., 2020). Indeed, overexpressed genes were enriched 3.9- (p < 1 × 10^−6^) and 2.0-fold (p < 1 × 10^−6^) for activated fibroblast gene signatures, as recently defined by two single-cell RNA-seq analyses of fibrotic lungs (Peyser et al., 2019 and Tsukui et al., 2020). Conversely, the overexpressed genes captured 39% and 20%, respectively, of the Peyser and Tsukui gene sets (Figure 3B). Among the most highly overexpressed genes were those associated with wounding-induced ECM remodeling (e.g., *Mmp10*, *Mmp3*, *Loxl1*, *Col8a2*, *Tnc*, as well as *Col1a1*, as expected) (Bonnans et al., 2014). By contrast, however, there was no change in the expression of H3K27me3-marked genes associated with leukocyte chemotaxis or expansion, including *Cxcl9*, *Cxcl10*, and *Csf1*, with the lack of *Csf1* induction potentially explaining the *Ezh2* cKO decidua’s failure to accumulate the macrophages noted above. Importantly, we observed no underexpression of 20 key markers and regulators of decidualization (e.g., *Prl8a2*, *Bmp2*, *Pgr*, and *E2f8*; Table S3) (Cha et al., 2012). qRT-PCR analysis confirmed the RNA-seq data for highly differentially expressed genes and revealed that these differences were also apparent in mice treated with P4 (Figure S5A). Indeed, P4 administration also had no major effect on the decidual expression of putative P4 target genes (Wetendorf and DeMayo, 2012), consistent with serum levels of endogenous P4 already being high in early gestation (Figure S5B). Together, these observations indicated that EZH2 limits fibroblast activation/myofibroblast formation within the decidua and provided further evidence that pregnancy failure in *Ezh2* cKO mice was not due to spontaneous decidualization failure but rather to a superimposed stromal cell reaction of the kind typically associated with wound healing.

### Overactivation of wound-healing-associated TGF-β responses in EZH2-deficient DSCs

The above observations also suggested the potential involvement of TGF-β in the *Ezh2* cKO phenotype given the central role of TGF-β in wounding-induced stromal cell responses, including its potent ability to induce fibroblast activation and fibrogenic ECM remodeling (Hinz et al., 2012; Meng et al., 2016). Importantly, TGF-β1 is highly expressed in the mouse decidua, and genetic ablation of the type I TGF-β receptor (TGFβRI) causes multiple decidual abnormalities (Fang et al., 2020; Ni and Li, 2017; Peng et al., 2015). However, fibroblast activation and fibrogenic ECM remodeling are not normally evident within decidual tissue. Thus, to evaluate whether EZH2 suppresses these specific features of the TGF-β response within DSCs, we purified DSCs from control and *Ezh2* cKO mice and performed RNA-seq to determine the scope of the cells’ respective transcriptional changes following TGF-β exposure.

**Figure 4.**
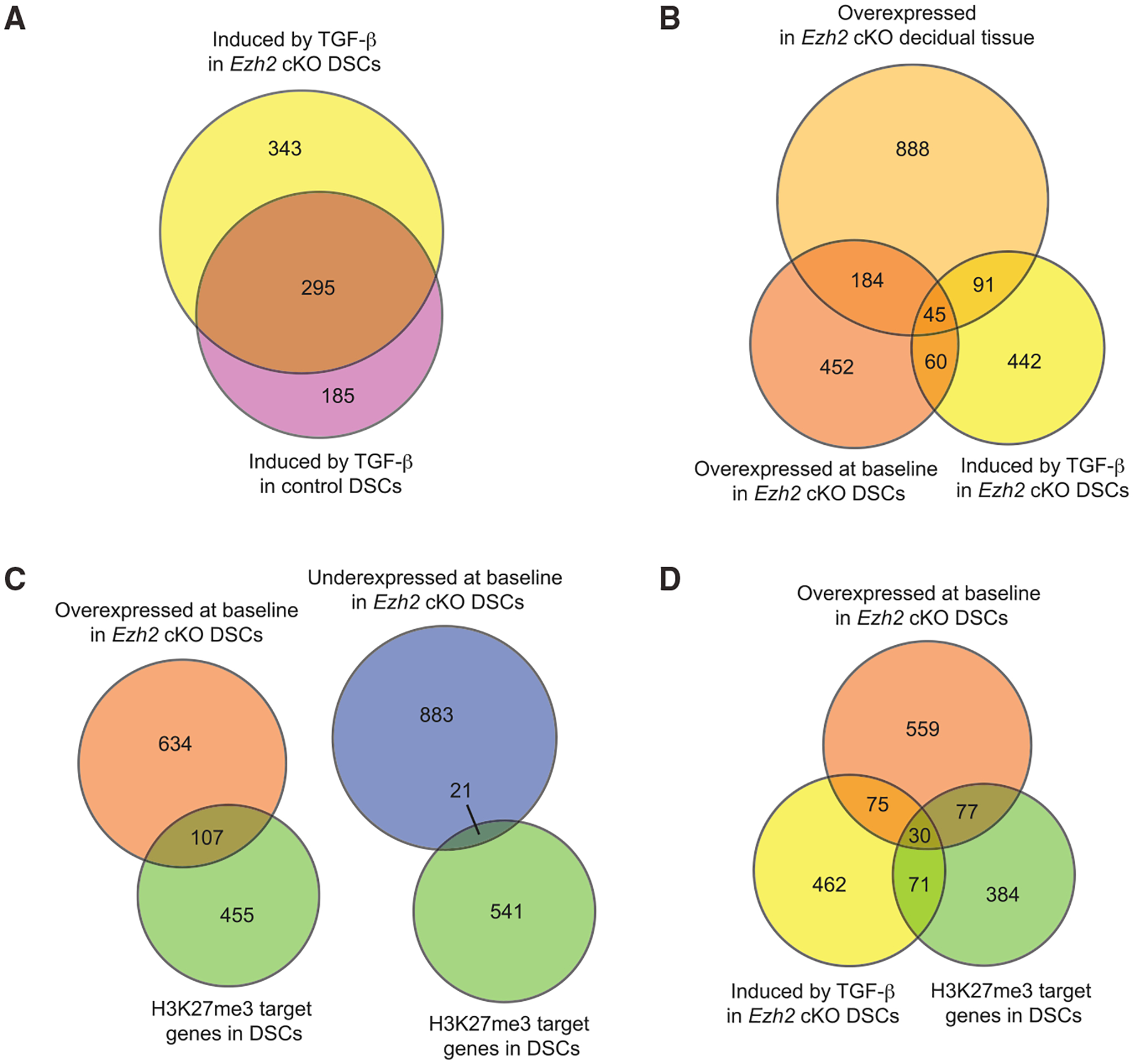
H3K27me3-mediated gene silencing and TGF-β activity are closely linked in DSCs Proportional Venn diagrams illustrating features of an RNA-seq analysis of control and *Ezh2* cKO DSCs. The cells were purified from E7.5 implantation sites of mice not given P4 and were cultured for 24 h with or without 2 ng/mL TGF-β. n = 6 biological samples per group, with each sample split between the TGF-β treated and untreated conditions allowing for paired analysis. As with Figure 3, we only considered protein-coding genes that were expressed more than 30 RPKM (12,096 total, with the RPKM for *Actb* being about 130,000). In this case, application of the expression threshold reduced the size of the H3K27me3 target-gene set in DSCs to 562. The threshold for differential expression was FDR < 0.05. Data for whole decidual tissue are from the RNA-seq experiment described in Figure 3; H3K27me3 target genes in DSCs are those previously identified by Nancy et al. (2018). “Baseline” gene expression by DSCs refers to that seen in the absence of TGF-β treatment. See the main text for the hypergeometric-test-determined p values indicating whether overlapping gene sets are significantly larger or smaller than what would be expected by chance. (A) Degree of overlap between genes induced by TGF-β in control and *Ezh2* cKO DSCs. (B) Degree of overlap between genes overexpressed atbaseline in*Ezh2*cKO whole decidual tissue, genes overexpressed at baseline in *Ezh2* cKO DSCs, and genes induced by TGF-β in *Ezh2* cKO DSCs. (C) Degree of overlap between H3K27me3 target genes and genes either overexpressed or underexpressed at baseline in *Ezh2* cKO DSCs. (D) Degree of overlap between genes that are overexpressed in *Ezh2* cKO DSCs at baseline, genes induced by TGF-β in *Ezh2* cKO DSCs, and H3K27me3 target genes.

TGF-β induced 638 protein-coding genes in EZH2-deficient DSCs versus 480 in control DSCs (with a 295 gene overlap), demonstrating that EZH2 deficiency shifts the responsiveness of DSCs to TGF-β at the genome-wide level (p < 0.0001, Fisher’s exact test; Figure 4A; Table S4). Within the 638 genes (henceforth referred to as TGF-β target genes), activated fibroblast signature genes were highly overrepresented, with 5.8- and 3.9-fold enrichments of the Peyser (p < 1 × 10^−7^) and Tsukui (p < 1 × 10^−17^) gene sets, respectively. Moreover, TGF-β target genes were 2.7-fold overrepresented within the set of genes with a higher expression in EZH2-deficient DSCs at baseline (i.e., when neither EZH2-deficient nor control DSCs were treated with TGF-β; p < 1 × 10^−20^) as well as 2.6-fold overrepresented within the set of genes with a higher expression in whole EZH2-deficient decidual tissue (p < 1 × 10^−24^; Figure 4B). Together, these observations suggested that the loss of gene silencing in EZH2-deficient DSCs increases TGF-β activity in the decidua, with its effects on DSCs in turn potentially explaining the *Ezh2* cKO decidua’s activated fibroblast signature.

We further noted that H3K27me3 target genes were 3.1-fold overrepresented among those with a higher expression at baseline in EZH2-deficient DSCs (p < 1 × 10^−26^; Figure 4C) and 2.0-fold underrepresented among those with lower expression (p < 1 × 10^−3^; Figure 4C). Conversely, 19.2% of all H3K27me3 target genes were expressed at higher levels in *Ezh2* cKO DSCs at baseline but only 3.7% were expressed at lower levels. These observations are in accord with our whole-tissue analysis (Figure 3A) and confirmed that a major function of EZH2 in DSCs is to inhibit gene expression. Importantly, H3K27me3 target genes were highly overrepresented among TGF-β target genes, as they constituted 15.8% of the TGF-β target-gene set (101 genes out of 638 genes total, which is a 3.5-fold enrichment; p < 1 × 10^−29^) and almost one-third (28.6%) of the TGF-β target genes that were upregulated at baseline in EZH2-deficient DSCs (30 genes out of 105 genes total, which is a 4.7-fold enrichment; p < 1 × 10^−12^) (Figure 4D). Provocatively, the most TGF-β-inducible gene on the list of 30 was *Nrep*, which encodes a fibrosis-associated factor (P311) that increases TGF-β translation (Duan et al., 2019; Yao et al., 2015) (Table S4). *Nrep* was expressed 6.7-fold higher at baseline in EZH2-deficient DSCs, and TGF-β exposure increased its expression 8.3-fold more. *Nrep* was also overexpressed 1.6-fold in whole *Ezh2* cKO decidua and, as discussed below, is downregulated in ESCs as they decidualize, together with 19 other genes on the list of 30 (Table S4). Together, these data revealed a strong bias of H3K27me3-mediated silencing toward TGF-β-inducible genes with constrained expression within DSCs, with the silencing of *Nrep* additionally suggesting a mechanism for limiting the generation of TGF-β activity within the decidua.

### Genes with greater TGF-β inducibility tend to be transcriptionally silenced during decidualization

**Figure 5.**
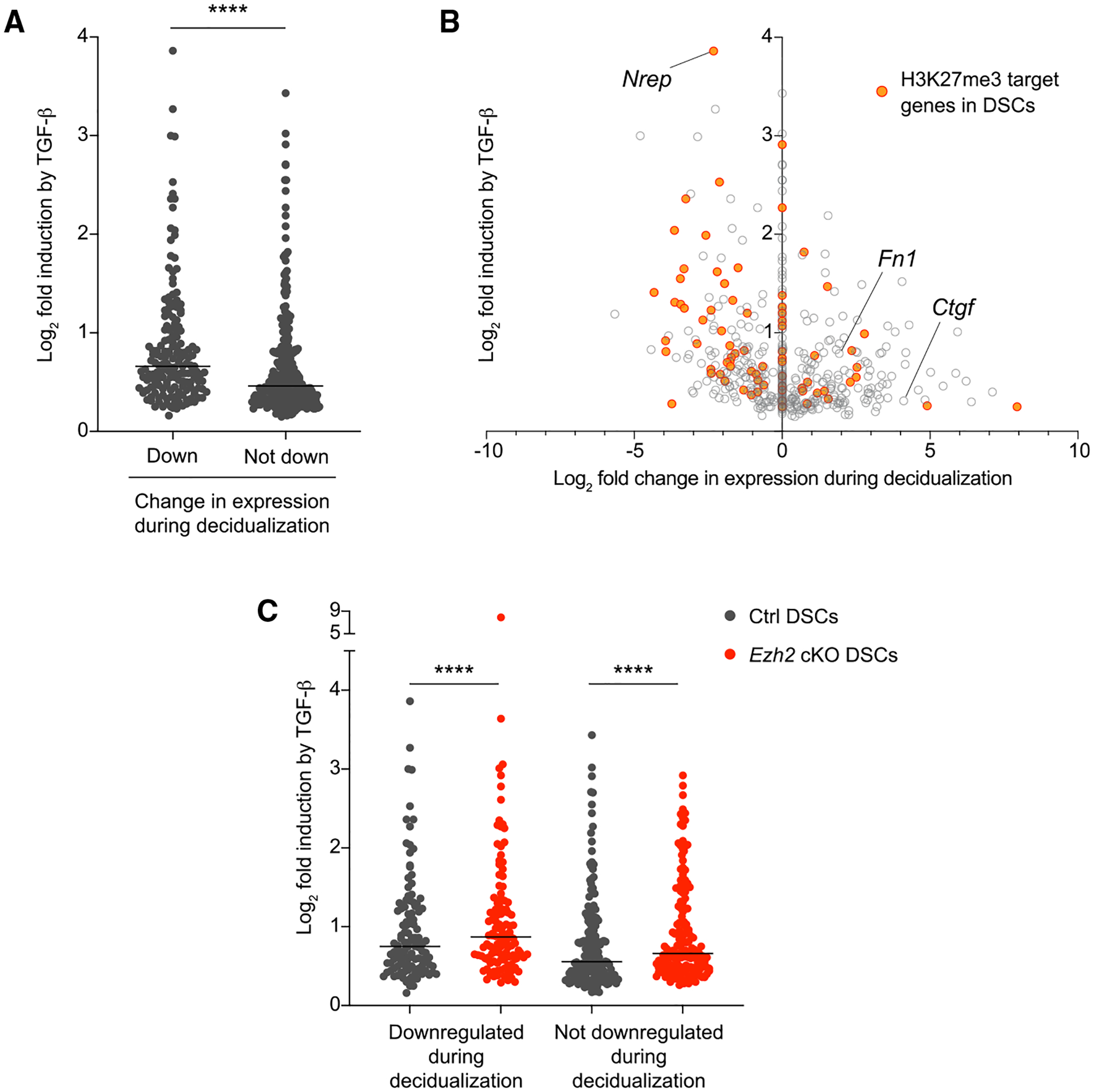
Higher inducibility of TGF-β target genes in DSCs predicts transcriptional downregulation during decidualization (A and B) Comparisons between the fold induction of TGF-β target genes in control E7.5 DSCs following TGF-β treatment and the respective change in these genes’ expression that occurs with decidualization, i.e., when comparing E7.5 DSCs with undecidualized uterine stromal cells (USCs). Data for TGF-β inducibility are from the RNA-seq experiment described in Figure 4; data for gene expression differences between DSCs and USCs are from Nancy et al. (2018). For (A), we grouped the genes according to whether they were downregulated during decidualization (i.e., they showed significantly [FDR < 0.05] lower expression in DSCs compared with in USCs; n = 170) or not (i.e., they showed significantly higher expression or no significant change in expression compared with USCs; n = 293). Lines denote median TGF-β inducibility. ****p < 0.0001, Mann-Whitney test. For (B), we plotted fold induction in explicit relationship to the fold change in expression between DSCs and USCs, with genes showing no significant difference between DSCs and USCs plotted at 0.0. (C) Fold induction of the set of TGF-β target genes shared by control and *Ezh2* cKO DSCs (see Figure 4A). We only analyzed the 285 genes (of the total 295) for which we had DSC versus USC expression data. As in (A), we grouped the genes according to whether they are downregulated in E7.5 DSCs compared with in USCs (n = 119), or not (n = 166). Lines denote median TGF-β inducibility. ****p < 0.0001, Wilcoxon signed-rank test.

To gain insight into why only certain TGF-β target genes were marked for transcriptional silencing in DSCs, we cross-correlated TGF-β responsiveness, as assessed above, to the gene expression changes that occur in ESCs during decidualization. This analysis took advantage of our previously published RNA-seq-based comparison of E7.5 DSCs and the (undecidualized) stromal cells purified from the uteri of P4-treated non-pregnant mice (generically termed uterine stromal cells, or USCs, but pre-dominantly comprised of ESCs and a small number of myometrial fibroblasts [J.S. and A.E., unpublished data]) (Nancy et al., 2018). Notably, for the 463 out of 480 total TGF-β target genes in control DSCs for which we had expression data from that previous study, 170 (36.7%) were expressed at lower levels in DSCs compared with in USCs, 182 (39.3%) were expressed at higher levels, and 111 (24.0%) showed no change in expression. Thus, being a TGF-β target gene per se did not determine whether a gene was up- or downregulated during decidualization. However, the genes that were downregulated during decidualization showed markedly greater fold-inducibility by TGF-β compared with those that were not (Figure 5A). The downregulated group was also greatly enriched for H3K27me3 targets (24.7% compared with 11.3%, p = 0.002 by Fisher’s exact test), including *Nrep* (Figure 5B). Thus, high sensitivity to induction by TGF-β appeared as a determinant of gene downregulation during decidualization, in part mediated by H3K27me3 promoter accrual. Interestingly, TGF-β targets that were upregulated during decidualization included the classic TGF-β targets *Fn1* and *Ctgf* (Figure 5B). The promoters of these two genes do not accrue H3K27me3 during decidualization (Nancy et al., 2018), and their encoded proteins, fibronectin and connective tissue growth factor, are wound-healing-associated ECM components as well as fibroblast activation markers (Bonnans et al., 2014; Hinz, 2015; Peyser et al., 2019; Tsukui et al., 2020). Thus, even while silencing a large number of TGF-β-inducible fibroblast activation markers upon decidualization, ESCs induce the expression of others.

As expected, a similar relationship between TGF-β inducibility and differential expression between DSCs and USCs was evident for the genes identified as TGF-β targets in *Ezh2* cKO DSCs (Figures S5C and S5D). In addition, when we analyzed the genes that were TGF-β targets in both control and *Ezh2* cKO DSCs so that we could performed paired analyses, we found that they were more inducible in *Ezh2* cKO DSCs independent of whether they were downregulated or not during decidualization (Figure 5C). This observation was consistent either with EZH2 deficiency in DSCs somehow increasing the signal strength generated by a fixed concentration of TGF-β or with generating increased autocrine TGF-β activity over the 24 h *ex vivo* culture period, a possibility in turn consistent with the increased expression of *Nrep* in *Ezh2* cKO DSCs.

### Inhibition of TGF-β signaling reduces fibroblast activation in the *Ezh2* cKO decidua and improves pregnancy outcomes in *Ezh2* cKO mice

**Figure 6.**
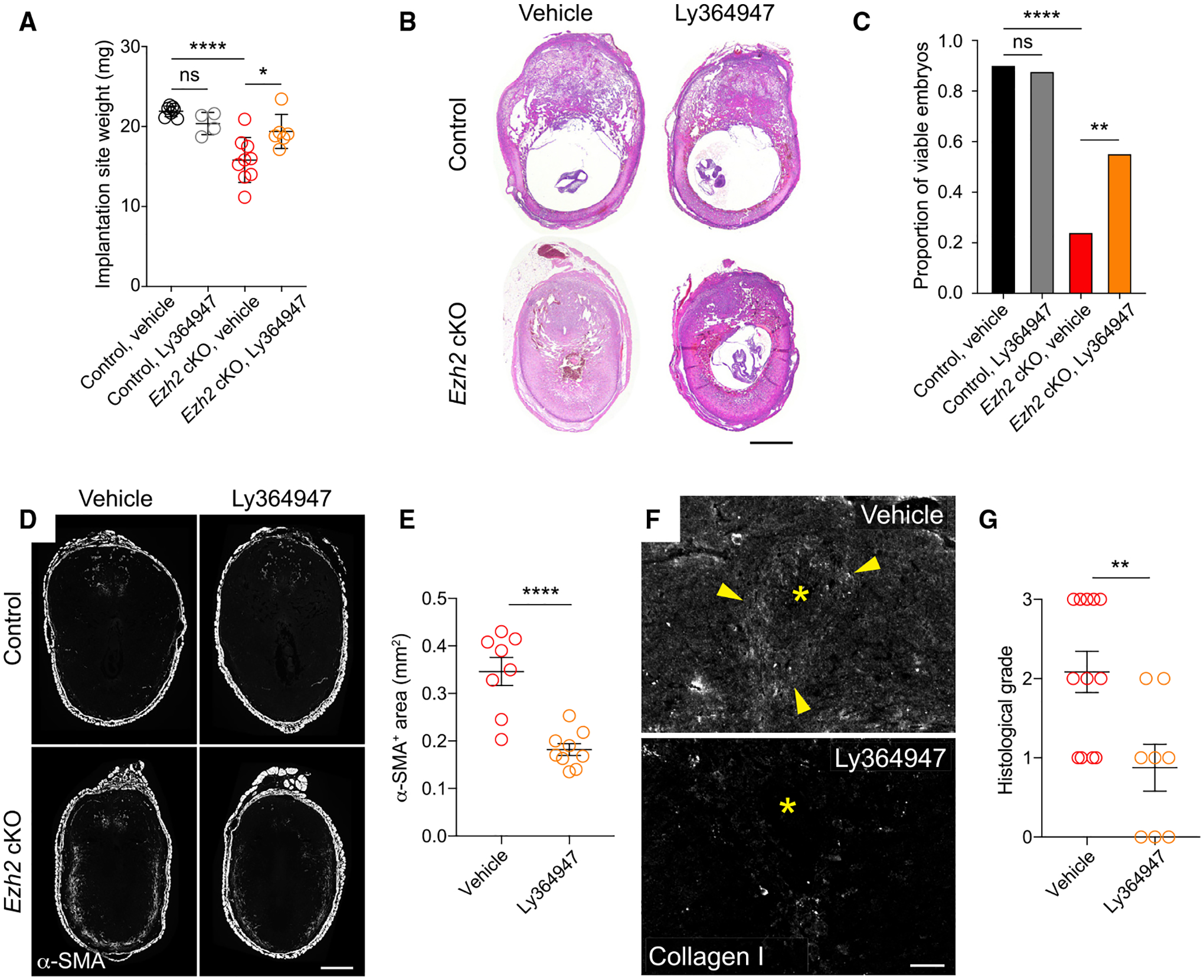
Inhibition of TGFβRI signaling during pregnancy reduces fibroblast activation in the *Ezh2* cKO decidua and increases embryo viability at mid-gestation Control and *Ezh2* cKO mice were injected daily with 2 mg P4 and either the TGFβRI inhibitor Ly364947 or vehicle (20% DMSO) starting on E5.5. (A) Implantation site weights on E7.5. Data show mean ± SEM of the average implantation site weight for each mouse. n = 6–9 mice/group; *p < 0.05; ****p < 0.0001, one-way ANOVA with Sidak’s multiple comparisons test. (B and C) Representative H&E staining of E9.5 implantation sites (B) and quantification of live embryo proportions, as determined histologically (C). Data represent a total of n = 42 embryos from n = 5 vehicle-treated control mice, n = 25 embryos from n = 4 Ly364947-treated control mice, n = 50 embryos from n = 6 vehicle-treated *Ezh2* cKO mice, and n = 47 embryos from n = 8 Ly364947-treated *Ezh2* cKO mice. ****adjusted p value [p_adj_] < 0.0001; **p_adj_ < 0.01, Fisher’s exact test with Bonferroni correction. Scale bar, 500 μm. (D and E) Immunofluorescence detection (D) and morphometric quantification (E) of anti-mesometrial decidual α-SMA expression in E7.5 implantation sites (mean ± SEM; n = 4 mice/group, 2–3 implantation sites quantified per mouse; ****p < 0.0001, Student’s t test). Scale bar, 500 μm. (F and G) Immunofluorescence detection (F) and quantification (G) of type I collagen accumulation around embryos on E7.5 (n = 5 mice/group; 1–3 implantation sites quantified per mouse, **p < 0.01, Student’s *t*-test). Quantification was performed by a blinded observer who scored photomicrographs for collagen I staining intensity on a scale of 0 (no peri-embryonic collagen) to 3 (copious peri-embryonic collagen). Scale bar, 100 μm.

To functionally assess whether TGF-β activity contributed to the *Ezh2* cKO phenotype, we treated pregnant animals with Ly364947, a pregnancy-compatible small-molecule inhibitor of TGFβRI (Peng et al., 2005). To avoid disrupting early decidualization, we started giving the inhibitor on E5.5 and treated until sacrifice. The mice also received daily P4 injections starting on E5.5 to render irrelevant any effects of Ly364947 on ovarian function. Importantly, the dosing of Ly364947 administration in our experiments did not affect embryo viability nor implantation site weights in control mice at E9.5 (Figures 6A–6C), unlike the modest reduction in implantation site weights seen on E9.5 in *Pgr*^*cre/+*^ *Tgfbr1*^*fl/f*^ mice with genetic ablation of TGFβRI in the uterus. On the other hand, Ly364947 administration slightly reduced decidual expression of *Il15*, a highly downregulated gene in the *Pgr*^*cre/+*^ *Tgfbr1*^*fl/f*^ decidua (Figure S6A). These data suggested that Ly364947 only partially inhibits TGF-β signaling in the decidua. Nonetheless, Ly364947 treatment normalized *Ezh2* cKO implantation site weights on E7.5 and increased the proportion of *Ezh2* cKO mice with normal-appearing implantation sites and viable embryos on E9.5 (Figures 6A–6C). These effects were associated with reduced α-SMA expression in the anti-mesometrial decidua and with reduced amounts of peri-embryonic collagen (Figures 6D–6G). Together, these observations suggested that much of the *Ezh2* cKO phenotype was due to increased TGF-β activity within the decidua.

### EZH2 deficiency in DSCs unmasks only select elements of a classical wound-healing response when the decidua is surgically wounded

Previously, we demonstrated that embryo-free artificial decidua minimally accumulate α-SMA^+^ cells when surgically wounded, a result formally demonstrating that the underlying mechanism was intrinsic to the decidua itself (Nancy et al., 2018). To determine whether this finding was due to EZH2-mediated gene silencing in DSCs, and to gauge the overall breadth of the wounding response unmasked by EZH2 deficiency and the extent to which it mimicked the broad Ezh2 cKO phenotype, we assessed the localized response of pregnant *Ezh2* cKO mice to surgical wounds created within the decidua. The wounds were generated on E6.5 by piercing implantation sites with a 26G needle, through which we injected GFP-expressing lentiviruses so that we could identify the wound sites 48 h later by anti-GFP immunostaining.

**Figure 7.**
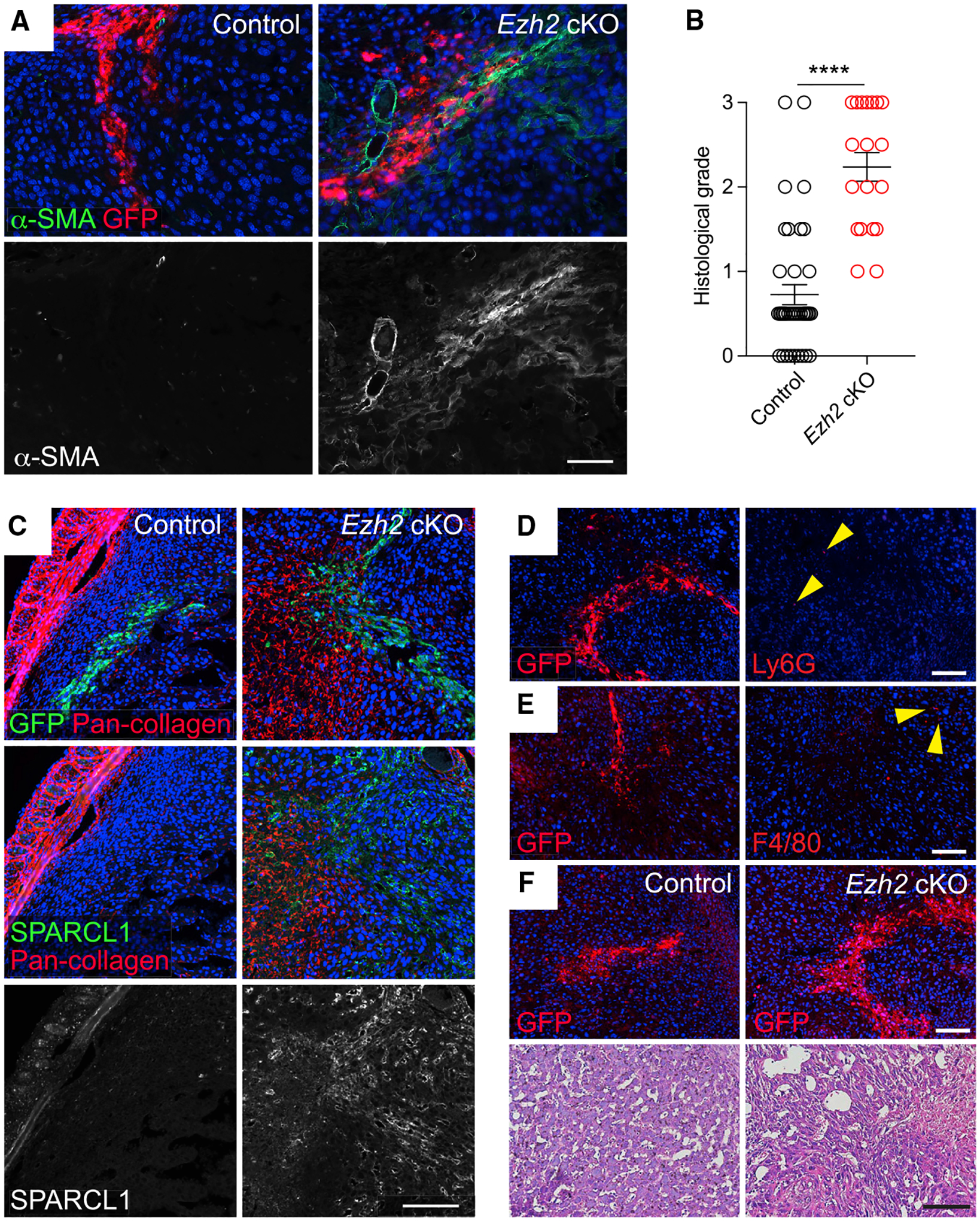
Wounding induces myofibroblast formation, collagen deposition, and TGF-β target-gene expression in *Ezh2* cKO but not control decidua Implantation sites of E6.5 control and *Ezh2* cKO mice were wounded by piercing them with a fine needle, through which we injected lentiviruses engineered to constitutively express GFP. The mice then received daily P4 injections until their sacrifice 48 h later. (A) Immunofluorescence detection of α-SMA^+^ and GFP, which marks wound sites. Representative images from n = 7 mice/group (2–8 wound sites/mouse). Scale bar, 50 μm. (B) Quantification of α-SMA expression. A blinded observer scored the quantity of α-SMA expression at wound sites from a scale of 0 (no α-SMA^+^ cells) to 3 (copious α-SMA^+^ cells) (mean ± SEM; n = 6 mice/group, 2–8 wound sites/mouse; ***p < 0.0001, Student’s t test). (C) SPARCL1 and total collagen expression at wound sites, detected by immunofluorescence (SPARCL1) and a fluorescent pan-collagen reagent (total collagen; see STAR Methods). Serial sections were stained as indicated. Representative images from n = 7 mice/group (2–5 wound sites/mouse). Scale bar, 100 μm. (D and E) Immunofluorescence detection of Ly6G^+^ neutrophils (D) and F4/80^+^ macrophages (E) at GFP^+^ wound sites within the *Ezh2* cKO decidua. Serial sections were stained as indicated. Very few neutrophils or macrophages (arrowheads) are present. Representative images from n = 3 mice (2–3 wound sites/mouse) for the Ly6G staining and n = 5 mice (1–3 wound sites/mouse) for the F4/80 staining. Scale bars, 50 μm. (F) H&E-stained wound sites from control and *Ezh2* cKO implantation sites. Serial sections were stained with GFP to identify wound sites (upper panels). Representative images from n = 4 mice/group (1–3 wound sites/mouse). Scale bars, white (immunofluorescence), 50 μm and black (H&E), 100 μm.

Strikingly, wound sites in *Ezh2* cKO but not control decidua showed α-SMA^+^ myofibroblast accumulation (Figures 7A and 7B) and collagen deposition (~20% occurrence; Figure 7C). These observations in turn implied the presence of TGF-β activity in the wound site, and to confirm this point, we took advantage of our RNA-seq analysis to identify *Sparcl1*, which encodes a protein that regulates ECM assembly (Sullivan et al., 2006), as a highly TGF-β-inducible gene in EZH2-deficient DSCs with high absolute expression once induced (Table S4; data not shown). Indeed, SPARCL1 expression was diffusely elevated throughout the decidua of non-wounded, vehicle-treated *Ezh2* cKO mice but was largely absent from control decidua and greatly reduced in the decidua of Ly364947-treated *Ezh2* cKO mice (Figure S6B). As shown in Figure 7C, however, SPARCL1 expression was also super-induced at the wound sites of Ezh2 cKO decidua but was not expressed in the wound sites of control decidua.

Together, these data indicated that EZH2 deficiency within DSCs allowed the decidua to manifest a classical stromal response to wounding that recapitulated the overall phenotype of the *Ezh2* cKO decidua. Moreover, since we induced wounding at E6.5, 2 days after implantation, these data confirmed that the wound-healing phenotype of the non-wounded *Ezh2* cKO decidua reflected an intrinsic property of this tissue and was not merely the consequence of earlier developmental events such as altered signaling from the EZH2-deficient uterine epithelium at the time of implantation. On the other hand, wounded *Ezh2* cKO decidua, like wounded control decidua, did not display the non-stromal features of wound healing. Specifically, neutrophil accumulation, a characteristic early event post-wounding, was not apparent within *Ezh2* cKO decidual wounds (Figure 7D), even though neutrophils accumulated in wounded non-pregnant *Ezh2* cKO and control uteri (Figure S7), nor did *Ezh2* cKO decidual wounds accumulate macrophages (Figure 7E), similar to our prior results with wounded artificial decidua (Nancy et al., 2018). These observations thus also directly paralleled the overall *Ezh2* cKO decidual phenotype, in particular the lack of leukocyte accumulation within the portion of the decidua that immediately surrounds the conceptus, where trophoblasts are invading into the tissue (Figure S3). In addition, neither control nor *Ezh2* cKO decidual wounds showed obvious evidence of neovascularization, a cardinal feature of granulation tissue formation, although this was difficult to evaluate given the high intrinsic vascularity of the decidua (Matsumoto et al., 2002) and the wound-associated hemorrhage (Figure 7F). Consistent with their accumulation of myofibroblasts, however, *Ezh2* cKO decidual wounds were more disorganized than their control counterparts (Figure 7F). Together, these results suggested that the pregnancy phenotype of *Ezh2* cKO mice represents a partially unmasked, TGF-β-driven response to the wound generated by embryo implantation and/or a wound-like stimulus generated by embryonic/placental development and decidualization. However, the response appeared confined to the stromal compartment of the tissue, suggesting the existence of yet additional pathways that mitigate wound healing at the maternal-fetal interface.

## DISCUSSION

In this report, we identify a critical role for EZH2 in suppressing key features of wound healing within the early-gestation mouse decidua. Specifically, we find that *Ezh2* deletion in DSCs results in ectopic myofibroblast formation, peri-embryonic collagen deposition, and decidual gene expression profiles associated with fibroblast activation and fibrogenic ECM remodeling. These abnormalities caused a high rate of pregnancy failure by mid-gestation and, mechanistically, were explained in large part by the 2-fold effects of (1) derepression of TGF-β-inducible genes in DSCs and (2) increased ambient TGF-β activity within the decidua. Indeed, it is likely that these two effects are intertwined, since the derepression of *Nrep*, a highly TGF-β-inducible H3K27me3 target in DSCs, by itself would be expected to increase intra-decidual TGF-β activity. Moreover, the ectopic myofibroblasts in the *Ezh2* cKO decidua would be expected to promote TGF-β activation by generating mechanical tension (Hinz, 2015).

EZH2 has been previously linked to wound healing, fibrosis, and the regulation of TGF-β activity; however, its effects appear context specific. For instance, EZH2 and H3K27me3 levels decrease upon cutaneous wounding (Shaw and Martin, 2009), suggesting that the non-wounded skin might tonically suppress fibroblast activation in ways similar to what we observe in the decidua. Similarly, EZH2-mediated suppression of TGF-β activity is critical for avoiding abnormalities in bone development (Mirzamohammadi et al., 2016). On the other hand, EZH2 expression is associated with enhanced TGF-β-induced differentiation of pulmonary fibroblasts into myofibroblasts and, consequently, pulmonary fibrosis (Xiao et al., 2016) as well as with the ability of TGF-β to promote the migration and differentiation of atrial myofibroblasts (Song et al., 2019).

One possible explanation for these differences is that PRC2 target-gene selection, and thus PRC2 function within a given cell, is itself contextually programmed by TGF-β signaling. Specifically, we note that in the early decidua, where EZH2 is anti-fibrotic, both TGF-β1 and its close relative activin are expressed at high levels (Maurya et al., 2013; Ni and Li, 2017; Shooner et al., 2005). In addition, TGF-β and activin in part signal through the transcription factor SMAD3, which has recently been shown to physically interact with EZH2 (Andrews et al., 2019; Oliviero et al., 2016). Thus, we speculate that concomitant SMAD3/PRC2 activation may establish an anti-fibrotic state that allows certain TGF-β target genes to be selectively marked for transcriptional silencing. As suggested by our finding that greater fold-inducibility by TGF-β predicts that a gene will be downregulated during decidualization, we further speculate that such target-gene selection may be determined by the signal strength present at each locus at the initial stages of the decidualization process. It remains to be determined whether this kind of mechanism thus allows PRC2 to create what appears to be a molecular “mask” that silences pregnancy-adverse TGF-β target-gene expression while allowing for the expression of those TGF-β targets that presumably mediate the positive contributions of TGF-β to decidualization (Fang et al., 2020; Peng et al., 2015). Such mechanisms might control the influence of EZH2-over TGF-β-regulated wound healing and fibrosis in other contexts, including the tumor microenvironment. It is also that possible that the requirements for TGF-β signaling within the decidua become more critical after the immediate post-implantation period, in accord with pregnant *Pgr*^*cre/+*^ *Tgfbr1*^*fl/f*^ mice only showing severe defects starting on ~E9.5 (Peng et al., 2015). In this scenario, TGF-β targets might only need to be transiently silenced by H3K27me3, thus allowing pregnancy to progress past this key danger period.

Interestingly, SPARCL1, which we identified as highly TGF-β-inducible in EZH2-deficient DSCs, was diffusely expressed throughout the *Ezh2* cKO decidua, suggesting the diffuse induction of TGF-β activity. By contrast, α-SMA^+^ myofibroblasts only appeared in the decidual periphery, while collagen deposition was most prominent around the embryo. Thus, additional contextual factors likely contribute to these latter two defects. Given their location, the generation of myofibroblasts may be related to signals emanating from the myometrium or the mechanical tension generated by it, while peri-embryonic collagen deposition may be a response to the progressive tissue disruption caused by invasive trophoblasts. It is also possible that these features of the *Ezh2* cKO phenotype are regionalized due to additional pathways that modulate wound healing within the decidua. This latter possibility is consistent with our prior observation that *Ccl5*, which encodes the broadly acting inflammatory chemokine CCL5 (RANTES), is transcriptionally silenced in DSCs without evident H3K27me3 promoter accrual (Nancy et al., 2018) as well as with the lack of neutrophil and macrophage accumulation in the surgically wounded *Ezh2* cKO decidua. These additional pathways may include DNA methylation and H2AK119 monoubiquitination via polycomb repressive complex 1 (PRC1), which are both already known to be required for decidualization (Bian et al., 2016; Gao et al., 2012). The activity of these pathways in the early decidua may also explain why certain H3K27me3 targets, such as *Cxcl9*, *Cxcl10*, and *Csf1*, are not derepressed in *Ezh2* cKO DSCs. Similarly, the continued silencing of these H3K27me3 targets might be explained by the action of EZH1, which is expressed by DSCs (Nancy et al., 2018) and functions as an alternate enzymatic component of PRC2. Indeed, PRC2-EZH1 is able to maintain transcriptional silencing of PRC2 target genes in non-DSC cell types following loss of EZH2, despite the cells showing the same global reductions in H3K27me3 levels we documented for DSCs (Lavarone et al., 2019; Margueron et al., 2008).

Remarkably, scant attention has been given to the fact that embryo implantation and placentation do not trigger a wound-healing response within the decidua. Our work highlights the importance of this non-response, as we show that it is the consequence, at least in part, of an active program of gene silencing necessary for pregnancy success in mice. Indeed, the threat of endometrial fibrosis to pregnancy is underscored by the multiple pregnancy complications experienced by women with Asherman syndrome, an endometrial scarring disorder that typically arises following surgical manipulation of the uterus (Salazar et al., 2017). It is currently unclear whether humans inhibit decidual wound healing through the same mechanisms as mice, nor is it known whether disinhibited wound healing is a cause of human pregnancy complications. However, it is notable that human DSCs downregulate α-SMA expression as they differentiate from ESCs, which in humans constitutively express this marker (Nancy et al., 2018; Vento-Tormo et al., 2018). It is also remarkable that the monthly cycle of destruction and regrowth experienced by the human endometrium occurs without scar formation. Our results here suggest that this fibrosis-resistant state may be the consequence of the human endometrium being partially decidualized prior to the onset of menses.

### Limitations of the study

In our lineage-tracing experiment assessing the cell-type specificity of Pgr-cre-mediated recombination (Figure S1), we found that some endothelial cells and leukocytes in *Pgr*^*cre/+*^ mTmG mice expressed low levels of GFP without losing tdTomato signal. While this phenotype likely reflects the phagocytosis of GFP^+^ material from surrounding recombined cells (see Figure S1), we cannot formally exclude the possibility that the GFP^lo^ endothelial cells and leukocytes instead represent cells that recently recombined, began expressing GFP, and then died. Accordingly, we cannot formally rule out the possibility that such transient EZH2-deficient cells contributed to the *Ezh2* cKO phenotype. Second, it is possible that EZH2-deficient epithelial cells contributed to the *Ezh2* cKO phenotype, although the phenotype of surgically wounded *Ezh2* cKO decidua argues against this possibility. Third, the *Ezh2* cKO phenotype appears to be influenced by strain background since the phenotype we describe for *Ezh2* cKO mice on a C57BL/6 background was subtly different than the phenotype previously described for *Ezh2* cKO mice on mixed C57BL/6; 129Sv backgrounds (Fang et al., 2019; Nanjappa et al., 2019). Finally, we cannot exclude contributions from systemic or off-target effects of the TGFβRI inhibitor Ly364947 as explanations for the reduction in fibroblast activation or the increase in embryo viability seen in Ly364947-treated *Ezh2* cKO mice.

## STAR★METHODS

### RESOURCE AVAILABILITY

#### Lead contact

Further information and requests for resources and reagents should be directed to and will be fulfilled by the lead contact, Adrian Erlebacher (adrian.elebacher@ucsf.edu).

#### Materials availability

This study did not generate any new unique reagents.

#### Data and code availability

RNA-seq data sets have been deposited at GEO and are publicly available as of the date of publication. In addition, this paper analyzes existing, publicly available single cell RNA-seq datasets. Accession numbers for all analyzed datasets are listed in the key resources table. Microscopy data reported in this paper will be shared by the lead contact upon request. This paper does not report original code. Any additional information required to reanalyze the data reported in this paper is available from the lead contact upon request.

### EXPERIMENTAL MODEL AND SUBJECT DETAILS

#### *In vivo* animal studies

*Ezh2* cKO (*Pgr*^*cre/+*^ *Ezh2*^*fl/fl*^) and littermate *Pgr*^+/+^ *Ezh2*^*fl/fl*^ mice (referred to in the main text as “control” mice) were generated through multi-generational intercrossing of *Pgr*^cre/+^ mice (Soyal et al., 2005) on a C57BL/6 background (the gift of Francesco DeMayo, National Institutes of Health) with *Ezh2*^*fl/fl*^ mice (Su et al., 2003) on a C57BL/6 background (the gift of Alexander Tarakhovsky, The Rockefeller University). C57BL/6J and mTmG (B6.129(Cg)-*Gt(ROSA)26Sor*^*tm4(ACTB-tdTomato,-EGFP)Luo*^/J) mice (Muzumdar et al., 2007) were purchased from The Jackson Laboratory. With irrelevance to the present study, these latter mice were modified so that sequences encoding ovalbumin-derived peptides were inserted into the GFP cassette. They were intercrossed with *Pgr*^cre/+^ mice to generate the *Pgr*-cre mTmG mice used in the lineage tracing experiments. 8–12 week old female mice were used for all experiments described in the paper. Male C57BL/6J breeder mice between 8 weeks and one year of age were used to impregnate experimental female mice. All animals were maintained in specific pathogen-free animal barrier facility, under ambient temperature and under a standard light-dark cycle. All experiments were approved by the University of California, San Francisco Institutional Animal Care and Use (IACUC) committee under protocol number AN178689.

#### Cell lines

The 293FT cell line (Thermo Fisher) was grown in D-MEM (high glucose) supplemented with 10% fetal bovine serum, 0.1 mM MEM non-essential amino acids, 6 mM L-glutamine, 1 mM sodium pyruvate, 1% penicillin-streptomycin, and 500 μg/mL Geneticin.

### METHOD DETAILS

#### Breeding and pregnancy outcome studies

To assess the overall fertility of *Ezh2* cKO female mice, 8–12-week old female virgins were singly housed with male C57BL/6J mice of proven fertility for a 10-week period, and the number of litters delivered and their sizes were recorded. To study pregnancy outcomes, 8–12-week old female mice were housed overnight with C57BL6/J males of proven fertility. Copulation plugs were assessed the following morning, with copulation plug formation denoting embryonic day 0.5 (E0.5) of pregnancy. As delineated in the main text and figure legends, some mice received 2 mg P4 in 100 μL sesame seed oil daily via subcutaneous injection starting on E5.5. Implantation site weights were determined on individually dissected implantation sites and then averaging these weights across the litter. To prepare E5.5 uteri for whole mount photography, mice were intravenously injected with 100 μL PBS containing 1% w/v Chicago Sky Blue 6B dye (Sigma) 5 min prior to sacrifice. Whole mount photographs were taken with a Nikon COOLPIX P600 digital camera.

#### Ly364947 treatments

The TGFβRI inhibitor Ly364947 (Selleckchem) was reconstituted in DMSO at 1 mg/mL upon arrival and stored for not more than one month at −20°C. Pregnant control and *Ezh2* cKO mice received daily intraperitoneal injections of 20 μg/mouse Ly364947 in 100 μL 20% DMSO or vehicle control (100 μL of 20% DMSO) starting on E5.5 until sacrifice. All mice concomitantly received daily subcutaneous injections of 2 mg P4 in sesame seed oil to prevent any potential direct or indirect effects of TGFβRI inhibition on P4 production by the ovary.

#### Lentiviral preparation

The FG12 viral vector (Addgene, no. 14884) containing a GFP insert was used to generate VSVG-pseudotyped lentiviral particles by concomitant transfection with psPAX2 (Addgene, no 12260) and pMD2G (Addgene, no 12259) into 293FT cells (Thermo Fisher) using the Lipofectamine 2000 Transfection Reagent (Thermo Fisher). Lentiviral supernatant was concentrated by spinning at 20,000*g* for 90 min in a Lynx4000 centrifuge and functional titer was assessed via infection of 293FT cells by serial dilution and flow cytometric analysis of the fraction of GFP^+^ cells on a BD LSRFortessa cytometer. The titer of the virus used for experiments was 1.5×10^8^ infectious particles/mL.

#### Wounding experiments

For wounding experiments on pregnant animals, mice were mated as described above. On E6.5 of pregnancy, one horn of the uterus was surgically exposed and implantation sites were pierced with a 26-gauge beveled needle (Hamilton 7758–04) attached to a syringe (Hamilton Gastight 1702), through which 0.5–1 μL virus was injected/wound. Each implantation site was wounded multiple times, receiving a total of 2–5 μL virus. Between 3 and 5 implantation sites were wounded per uterine horn. 2 mg P4 was injected subcutaneously on the day of surgery and 24 h afterward to render irrelevant any potential loss of ovarian function caused by the surgery. The mice were sacrificed on E8.5. For wounding experiments on non-pregnant animals, one horn of the uterus was surgically exposed and pierced with a needle-fitted Hamilton syringe, which was then used to make a longitudinal scratch along the inner surface of the endometrium. This technique was repeated several times per horn. The contralateral horn served as the non-wounded control. 2 mg P4 was injected subcutaneously 6 h and 24 h after surgery, and mice were sacrificed 48 h after surgery. Surgeries were performed according to institutional best practice as specified by the IACUC animal welfare protocol, including anesthesia, analgesia, and post-operative monitoring.

#### Immunohistochemistry and image analysis

Dissected uteri were fixed overnight at 4°C in 4% paraformaldehyde/PBS, embedded in paraffin (Paraplast X-TRA; Thermo Fisher Scientific), and sectioned at 5 μm. H&E staining was performed using routine methods. For immunofluorescence analysis, deparaffinized slides were first placed in methanol containing 3% H_2_O_2_ for 20 min. Depending on the primary antibody (see Table S5 for antibody sources, dilutions, and retrieval methods), slides then underwent antigen retrieval either by incubation with 1 mg/mL trypsin in PBS for 13 min at 37°C, or by boiling in 0.01 M citric acid (pH 6.0) or TE (0.01 M Tris Base, 0.01 M EDTA, 0.05% Tween 20, pH 9.0) in a decloaking chamber (BioCare Medical). Slides were then incubated for 1 h at room temperature in blocking buffer (3% BSA, 3% donkey serum, 0.4% Triton X-100 in PBS), then at 4°C overnight with primary antibody in PBS containing 1% BSA and 0.4% Triton X-100. Signal amplification, when employed, entailed incubation with horseradish peroxidase (HRP)-conjugated secondary antibodies (HRP-conjugated donkey anti-rabbit IgG, HRP-conjugated donkey anti-rat IgG, and HRP-conjugated donkey anti-goat IgG, all from Jackson ImmunoResearch Laboratories), biotin-tyramide amplification (PerkinElmer), and final incubation with streptavidin-conjugated Alexa Fluor 488 or Alexa Fluor 594 (Jackson ImmunoResearch Laboratories), as previously described (Nancy et al., 2012). Alexa Fluor 594 and Alexa Fluor 488-conjugated anti-rat IgG (Jackson ImmunoResearch Laboratories) were used as secondary antibodies in cases where amplification was not employed. Sections were then incubated with TrueBlack Lipofuscin Autofluorescence Quencher (Biotium) per manufacturer specifications and counterstained with DAPI. For pan-collagen staining, a Cy3-conjugated Collagen Hybridizing Peptide (3Helix) was diluted to a final concentration of 2.5 μM in PBS containing 1% BSA and 0.4% Triton X-100 to obtain the staining solution. This solution was then heated at 80°C for 5 min and cooled to room temperature. Additional primary antibodies were then added, and the solution was applied to the slides for 4°C overnight incubation. Subsequent staining proceeded as described above.

Brightfield images were captured using a Nikon Eclipse Ci-L microscope (2× objective) fitted with a DS-Ri2 color camera, and brightness/contrast adjustments were performed in Adobe Photoshop (Adobe Systems) to lighten the background. To determine the proportion of viable embryos (Figure 6C), we assessed H&E-stained E9.5 implantation sites. Non-viable embryos were scored as those showing necrosis, large-scale hemorrhage, or the absence of an amniotic cavity with concomitant presence of a stunted embryo.

Fluorescent microscopy was performed using an AxioImager M2 and Zen software (Zeiss). Panoramic views were generated by software-automated tiling of images taken with the 10× objective. Conversion from color to black and white images, as well as brightness and contrast enhancements were performed in Adobe Photoshop, with the same set of manipulations applied equally to all images of a given experiment. One exception was made in the case of the Ly6G images presented in Figure S7, where high variability in background staining necessitated slightly different brightness and contrast adjustments. To make cells visualized by anti-CD3 and anti-CD45 immunostaining (Figure S3) visible in panoramic views, pixel intensities were dilated using the Maximum filter of ImageJ software (NIH, https://imagej.nih.gov/ij/) as previously described (Collins et al., 2009; Nancy et al., 2012).

To determine the α-SMA^+^ area within sections of E7.5 implantation sites, a 100 μm by 100 μm grid was overlayed on top of a panoramic image of an implantation site stained for α-SMA using Adobe Photoshop. Grid squares containing α-SMA^+^ DSCs (but not myometrial, endothelial, or epithelial cells) were counted as positive and totaled to produce the final decidual α-SMA^+^ area in mm^2^. To determine the histological grade of α-SMA intensity in areas of wounded decidua, slides were double immunostained for GFP and α-SMA, and images of wounded areas (identified by GFP immunostaining) were randomized and given to a blinded reviewer to score on a scale of 0 (no apparent α-SMA expression by DSCs) to 3 (copious α-SMA expression by DSCs). Expression of α-SMA by vascular smooth muscle cells was not counted as positive signal, and regions that were obviously necrotic or undecidualized were excluded from analysis. To determine the histologic grade of peri-embryonic type I collagen expression, images of embryos were taken, randomized, and given to a blinded reviewer to score on a scale of 0 (no apparent peri-embryonic type I collagen signal) to 3 (copious peri-embryonic type I collagen signal).

#### Immunoblotting

Immunoblotting was performed on tissues that were lysed using ice-cold RIPA buffer supplemented with Halt Phosphatase and Protease Inhibitor Cocktail (Thermo Fisher Scientific). The samples were spun at 3,000*g* to remove debris and 10 μg of sample was resolved on a 4–12% Bis-Tris gel (Thermo Fisher) using SDS-PAGE. Samples were transferred onto a PVDF membrane (Thermo Fisher) and blocked in 5% milk in PBS with 0.1% Tween 20 (PBS-T). Membranes were washed 3 times in PBS-T after blocking and between each subsequent step. Membranes were incubated overnight at 4°C with primary antibody against type I collagen (Abcam, 1:1000), GAPDH (EMD Millipore, 1:1000), H3K27me3 (Cell Signaling Technology, 1:000), or total H3 (Abcam, 1:000) diluted in 1% Bovine Serum Albumin in PBS-T with 0.05% sodium azide. Subsequently, membranes were incubated with species-specific HRP-conjugated antibodies (goat anti-rabbit (Abcam); rabbit ant-chicken (EMD Millipore)), developed with Clarity Western ECL Substrate (Bio-Rad), and visualized with a ChemiDoc Touch Imaging System (Bio-Rad).

#### Stromal cell purification and culture

Implantation sites at E7.5 were dissected to separate the decidua from overlying myometrium, and the decidua was enzymatically disaggregated with Liberase 3 (Roche), DNase-I (Roche), and trypsin for 15 min on ice, followed by 45 min at 37°C with periodic trituration. EDTA was subsequently added to a final concentration of 5 mM, and cells were incubated for a further 15 min at 37°C. After digestion, DSCs were separated with LD magnetic bead columns (Miltenyi Biotec) according to the manufacturer’s protocol. Selection was carried out using microbead-coupled antibodies toward red blood cells (Ter-119; Miltenyi Biotec) and leukocytes (CD45; Miltenyi Biotec), together with antibodies against epithelial cells (CD326; University of Iowa hybridoma bank) and endothelial cells (CD102; Biolegend, clone 3C4), which were rendered magnetic though secondary application of microbead-coupled goat anti-rat IgG (Miltenyi Biotec). After isolation, DSCs from each biological isolate were split into two wells and cultured for 24 h at 0.5 × 10^6^ cells/well in 1 mL SFM4CHO medium supplemented with MEM nonessential amino acids, HEPES buffer, penicillin-streptomycin, L-glutamine, and sodium pyruvate, with or without 2 ng/mL TGF-β1 (InvivoGen).

#### RNA isolation and RNA-Seq

RNA was isolated from DSCs using RNeasy Mini reagents and columns (QIAGEN) according to the manufacturer’s protocol. For RNA extraction from whole tissues, implantation sites at E7.5 were dissected to separate the decidua from overlying myometrium and each layer was washed in cold PBS and homogenized in TRIzol (Thermo Fisher). RNA was subsequently purified by chloroform extraction and isopropyl alcohol precipitation. DNase digestion and RNA cleanup was performed using the RNase-Free DNase kit (QIAGEN) according to the manufacturer’s instructions. Samples were submitted to the UCSF Functional Genomics Core and processed as follows: after a quality control test, a single-end 50-bp RNA-Seq library was prepared (for whole tissue RNA: TruSeq mRNA kit, Illumina; for DSC RNA: Smartseq/NexteraXT kit, Illumina), followed by sequencing on an Illumina HiSeq 4,000 system. For whole tissues, we sequenced *n* = 4 control and *n* = 3 *Ezh2* cKO decidual specimens, and *n* = 3 control and *n* = 3 *Ezh2* cKO myometrial specimens; for cultured DSCs, we sequenced *n* = 6 independent biological isolates each of control and *Ezh2* cKO cells, each split into replicates with or without TGF-β1 treatment. Sequencing provided 731 million total reads for whole tissue samples and 706 million total reads for DSC samples. An average of 84.5% whole tissue reads and 82.2% DSC reads aligned with the mouse genome (Ensembl Mouse GRCm38.87). Alignment was preformed using Splice-aware STAR aligner STAR_2.4.2a (for analysis of whole tissue RNA) or STAR_2.5.2b (for DSC RNA). Reads uniquely mapped to known mRNAs were used to identify genes with differential expression (*FDR* < 0.05) using the DESeq2 R package. For tissue datasets, two separate analyses were performed: one comparing control to *Ezh2* cKO decidua and one comparing control to *Ezh2* cKO myometrium. Decidua and myometrium were not compared to each other. For DSC datasets, two separate analyses were performed: one comparing control and *Ezh2* cKO DSCs both cultured without TGF-β1 (i.e., comparing their “baseline expression”), and one comparing, in a paired fashion, each control or *Ezh2* cKO sample cultured without TGF-β1 to its biological replicate cultured with TGF-β1. For all subsequent analyses of both whole tissue and DSC-derived samples, non-protein coding genes and genes expressed lower than 30 RPKM were excluded. BioVenn software was used to generate proportional Venn diagrams for Figures 3 and 4 (https://www.biovenn.nl). Comparisons to the set of H3K27me3 target genes in DSCs and to the differences in gene expression in DSCs compared to USCs employed the gene set defined by our previously published ChIP-Seq and RNA-seq analyses (GEO GSE105456) (Nancy et al., 2018). Comparisons to fibroblast activation gene signatures employed data from Peyser et al. (Peyser et al., 2019) and Tsukui et al. (Tsukui et al., 2020). The Peyser gene signature was the set of 49 genes listed in that publication’s Table 2; the Tsukui gene signature was the set of 261 genes identified as being differentially overexpressed in cell cluster #8.

#### qRT-PCR

Whole-tissue RNA from Ezh2 cKO and control mice was isolated using TRIzol (Thermo Fisher), and cDNA was synthesized from 1 μg RNA using an iScript cDNA Synthesis Kit (Bio-Rad). The PCR reaction was performed with EvaGreen dye (Biotium) using a CFX Connect or CFX384 Real-Time PCR Detection System (Bio-Rad) and analyzed with CFX Manager software (Bio-Rad). Primers were designed with NCBI Primer-Blast software and a test qRT-PCR reaction was performed on every primer pair using decidual cDNA to ensure no self-amplification and to verify the melt curves. PCR products were run out on an agarose gel to confirm the correct product length. The cycle threshold (Ct) value of the housekeeping gene *Actb* (β-actin) was subtracted from the Ct of the experimental gene to determine the ΔCt. Statistical analyses were performed using ΔCt values. Expression of a given gene relative to *Actb* was plotted using the formula 2^−ΔCt^. For a full list of primers, see Table S6.

#### Flow cytometry

Implantation sites at E7.5 were dissected to separate the decidua from overlying myometrium, and the tissues were enzymatically disaggregated as described in the stromal cell purification section. All analyses were performed using an LSR Fortessa cytometer (BD) and FlowJo software (BD). After forward and side scatter gating to exclude debris and doublets, cells were subsetted based on viability, followed by cell type identification using fluorochrome-conjugated monoclonal antibodies to CD45, CD102, EpCAM, Ly6G, Ly6C, F4/80, CD11b, CD11c, and MHCII (see key resources table for commercial sources, specific clones, and fluorochromes). GFP and tdTomato expression was detected on the Blue-B and YG-D channels, respectively.

### QUANTIFICATION AND STATISTICAL ANALYSIS

All statistical analyses except hypergeometric tests (i.e., Student’s *t* test, one-way ANOVA with Sidak’s multiple comparisons test, Mann-Whitney test, Wilcoxon signed-rank test, and Fisher’s exact test) were performed using GraphPad Prism. Hypergeometric tests were performed using an online calculator (https://systems.crump.ucla.edu/hypergeometric/index.php). For all scatterplots and bar graphs, the error bars display the mean ± SEM or the geometric mean ± geometric SD, as indicated in the respective figure legends. The number of samples analyzed per experiment, the number of independent experiments performed, and the p value are indicated in the respective figure legends. p values for hypergeometric comparisons between various gene sets are listed in the respective sections of the text.

## Supplementary Material

1

2

3

4

5

6

## Figures and Tables

**Table:** KEY RESOURCES TABLE

REAGENT or RESOURCE	SOURCE	IDENTIFIER
Antibodies		
anti-Alpha-smooth muscle actin AF488 (Host: rat; clone 1A4)	eBioscience	Cat#53-9760-82; RRID:AB_2574461
anti-CD3 (Host: rabbit; polyclonal)	Agilent	Cat#A045229-2; RRID:AB_2335677
anti-CD45 (Host: rabbit; polyclonal)	Abcam	Cat#ab10558; RRID:AB_442810
anti-E2F8 (A38411; Host: rabbit; polyclonal)	Signalway Antibody	Cat#32173
anti-Ezh2 (Host: rabbit; clone SP129)	Millipore Sigma	Cat#SAB5500102
anti-SPARCL1 (Host: goat; polyclonal)	R&D Systems	Cat#AF2836; RRID:AB_2195097
anti-Type I collagen (Host: rabbit; polyclonal)	Abcam	Cat#ab34710; RRID:AB_731684
Peroxidase AffiniPure Donkey Anti-Rabbit IgG (H + L)	Jackson ImmunoResearch	Cat#711-035-152; RRID:AB_10015282
Peroxidase AffiniPure Donkey Anti-Rat IgG (H + L)	Jackson ImmunoResearch	Cat#712-035-150; RRID:AB_2340638
Peroxidase AffiniPure Donkey Anti-Goat IgG (H + L)	Jackson ImmunoResearch	Cat#705-035-003; RRID:AB_2340390
Alexa Fluor® 488 AffiniPure Donkey Anti-Rat IgG (H + L)	Jackson ImmunoResearch	Cat#712-545-150; RRID:AB_2340683
Alexa Fluor® 594 AffiniPure Donkey Anti-Rat IgG (H + L)	Jackson ImmunoResearch	Cat#712-585-153; RRID:AB_2340689
anti-CD102 BV421 (Host: rat; clone 3C4)	BD Biosciences	Cat#740018; RRID:AB_2739790
anti-CD326 (Ep-CAM) PE/Cy7 (Host: rat; clone G8.8)	Biolegend	Cat#118216; RRID:AB_1236471
anti-CD45 BUV395 (Host: rat; clone 30-F11)	BD Biosciences	Cat#564279; RRID:AB_2651134
anti-CD11b PerCP/Cy5.5 (Host: rat; clone M1/70)	Biolegend	Cat#101227; RRID:AB_893233
anti-Ly6G APC (Host: rat; clone 1A8)	Biolegend	Cat#127614; RRID:AB_2227348
anti-F4/80 APC/Cy7 (Host: rat; clone BM8)	Biolegend	Cat#123117; RRID:AB_893489
anti-CD11c PE/Cy7 (Host: hamster; clone N418)	Biolegend	Cat#117318; RRID:AB_493568
anti-MHCII BV421 (Host: rat; clone M5/114.15.2)	Biolegend	Cat#107631; RRID:AB_10900075
anti-Ly6C AF700 (Host: rat; clone HK1.4)	Biolegend	Cat#128023; RRID:AB_10640119
anti-CD326 (Host: rat; clone G8.8)	University of Iowa Hybridoma Bank	Cat#G8.8
anti-CD102 (Host: rat; clone 3C4)	Biolegend	Cat#105602; RRID:AB_313195
CD45 MicroBeads, mouse	Miltenyi Biotec	Cat#130-052-301; RRID:AB_2877061
Anti-Rat IgG MicroBeads	Miltenyi Biotec	Cat#130-048-502; RRID:AB_244364
Ter119 MicroBeads	Miltenyi Biotec	Cat#130-049-901
anti-GAPDH (Host: chicken; polyclonal)	EMD Millipore	Cat#AB2302; RRID:AB_10615768
anti-H3K27me3 (Host: rabbit; clone C36B11)	Cell Signaling Technology	Cat#9733T; RRID:AB_2616029
anti-H3 (Host: rabbit; polyclonal)	Abcam	Cat#ab18521; RRID:AB_732917
anti-rabbit IgG HRP (Host: goat; polyclonal)	Abcam	Cat#ab6721; RRID:AB_955447
anti-chicken IgG HRP (Host: goat; polyclonal)	EMD Millipore	Cat#AP162P; RRID:AB_11212232
anti-GFP (Host: rabbit; polyclonal)	Novus Biologicals	Cat#NB600-308; RRID:AB_10003058
anti-Ly6G (Host: rat; clone 1A8)	BD Biosciences	Cat#551459; RRID:AB_394206
anti-F4/80 (Host: rat; clone C1:A3-1)	Cedarlane	Cat#CL8940AP; RRID:AB_10060355
Chemicals, peptides, and recombinant proteins		
Cy3-conjugated Collagen Hybridizing Peptide	3Helix	Cat#red300
Ly364947	Selleckchem	Cat#S2805; CAS:396129-53-6
Recombinant human TGF-b1	InvivoGen	Cat#rcyc-htgfb1
Progesterone	Millipore Sigma	Cat#P0130; CAS:57-83-0
Chicago Sky Blue 6B	Millipore Sigma	Cat#C8679; CAS:2610-05-1
TrueBlack Lipofuscin Autofluorescence Quencher	Biotium	Cat#23007
Lipofectamine 2000	Thermo Fisher	Cat#11668030
EvaGreen Dye	Biotium	Cat#31000
Dimethyl Sulfoxide	BioWorld	Cat#40470004-3; CAS:67-68-5
Alexa Fluor 594 Streptavidin	Jackson ImmunoResearch	Cat#016-580-084
Alexa Fluor 488 Streptavidin	Jackson ImmunoResearch	Cat#016-540-084
Geneticin Selective Antibiotic (G418 Sulfate)	Thermo Fisher	Cat#10131035; CAS:108321-42-2
TRIzol reagent	Thermo Fisher	Cat#15596026
RIPA Lysis Buffer, 10×	EMD Millipore	Cat#20-188
Fetal Bovine Serum	R&D Systems	Cat#S11150; Lot#B21019
DMEM High Glucose Medium	Thermo Fisher	Cat#11965092
Penicillin-Streptomycin (100×)	GenClone	Cat#25512
L-Glutamine 200 mM	Thermo Fisher	Cat#25030-081
MEM Nonessential Amino Acids	Corning	Cat#25-025-CI
SFM4CHO Medium	HyClone	Cat#SH30549.02
Halt^™^ Protease and Phosphatase Inhibitor Cocktail (100×)	Thermo Fisher	Cat#78440
Trypsin from porcine pancreas, 1 mg tablets	Millipore Sigma	Cat#T7168-20TAB; CAS:9002-07-7
Bovine Serum Albumin	Millipore Sigma	Cat#A2153-500G; CAS:9048-46-8
Triton X-100	Millipore Sigma	Cat#X100-500ML; CAS:9036-19-5
Tween 20	Millipore Sigma	Cat#P1379-1L; CAS:9005-64-5
Paraplast X-TRA	Millipore Sigma	Cat#P3808-1KG
EDTA 0.5M	Promega	Cat#V4233; CAS:6381-92-6
Tris Base	Thermo Fisher	Cat#BP152-1; CAS:77861
Citric Acid	Millipore Sigma	Cat#27487-250G-F; CAS:77-92-9
HEPES Buffer	Corning	Cat#25-060-CI
Sodium Pyruvate	GenClone	Cat#25-537
Donkey Serum	Millipore Sigma	Cat#S30-100ML; CAS:999999-99-4
Tyramide Signal Ampification Biotin Kit	Perkin Elmer	Cat#SAT700001KT
Paraformaldehyde	Millipore Sigma	Cat#158127-500G; CAS:30525-89-4
Sesame Seed Oil	Millipore Sigma	Cat#S3547-1L; CAS:8008-74-0
Deposited data		
RNA sequencing of control and *Ezh2* cKO whole tissue decidua at E7.5	Gene Expression Omnibus Database	This paper; GEO#GSE171723
RNA sequencing of control and *Ezh2* cKO decidual stromal cells treated with TGF-α1	Gene Expression Omnibus Database	This paper; GEO#GSE171724
Data from RNA sequencing of lung fibroblasts.	Laboratory of Lori Morton, Regeneron Pharmaceuticals	Peyser et al., 2019; GEO#GSE129605
Data from RNA sequencing of lung fibroblasts.	Laboratory of Dean Sheppard, University of California, San Francisco	Tsukui et al., 2020; GEO#GSE132771
Experimental models: Cell lines		
293FT Cell Line	Thermo Fisher	Cat#R70007
Experimental models: Organisms/strains		
Model organism: *Ezh2*^*fl*/*fl*^	Alexander Tarakhovsky, The Rockefeller University	Su et al., 2003
Model organism: *Pgr*^*Cre*/+^	Francesco DeMayo, National Institutes of Health	Soyal et al., 2005
Model organism: *mTmG*: (B6.129(Cg)-*Gt(ROSA)26Sor*^*tm4(ACTB-tdTomato,-EGFP)Luo*^/J)	The Jackson Laboratory	Cat#007676; RRID:IMSR_JAX:007676
Model organism: C57BL/6J: C57BL/6J	The Jackson Laboratory	Cat#000664; RRID:IMSR_JAX:000664
Oligonucleotides		
qRT-PCR primers	Elim Biopharm	Table S6
Recombinant DNA		
FG12 Lentiviral Vector	Addgene	Addgene; Cat#14884; RRID:Addgene_14884
psPAX2 Lentiviral Vector	Addgene	Addgene; Cat#12260; RRID:Addgene_12260
pMD2G Lentiviral Vector	Addgene	Addgene; Cat#12259;
Software and algorithms		
FlowJo	BD	https://www.flowjo.com/
GraphPad Prism	GraphPad	https://www.graphpad.com/
Online hypergeometric calculator	Graeber Lab, University of California, Los Angeles	https://systems.crump.ucla.edu/hypergeometric/index.php
ImageJ Software	National Institutes of Health	RRID:SCR_003070; https://imagej.nih.gov/ij/
CFX Manager Software	Bio-Rad	https://www.bio-rad.com/en-us/sku/1845000-cfx-manager-software?ID=1845000
NCBI Primer Blast software	National Institutes of Health	https://www.ncbi.nlm.nih.gov/tools/primer-blast/
Adobe Photoshop	Adobe Systems	https://www.adobe.com
Adobe Illustrator	Adobe Systems	https://www.adobe.com
ZEN Software	Zeiss	https://www.zeiss.com/
Other		
26 gauge beveled needle	Hamilton	Cat#7758-04
Hamilton Gastight syringe	Hamilton	Cat#1702
AxioImager.M2 fluorescent microscope	Zeiss	https://www.zeiss.com/microscopy/us/products/light-microscopes/axio-imager-2-for-biology.html
Nikon Eclipse Ci-L microscope	Nikon	https://www.microscope.healthcare.nikon.com/products/upright-microscopes/eclipse-ci-series

## Highlights

- EZH2 prevents implantation from triggering wound-healing responses in the uterus
- EZH2 prevents wound healing in the uterus by suppressing TGF-β activity
- PRC2-generated H3K27me3 marks transcriptionally silence TGF-β target genes
- Derepressed wound-healing responses in the uterus lead to pregnancy failure
