# Supplementary material for: Gene silencing by EZH2 suppresses TGF-β activity within the decidua to avert pregnancy-adverse wound healing at the maternal-fetal interface
Source: Cell Rep. Author manuscript; Available in PMC 2022 Feb 11. (PMC8833843; doi:10.1016/j.celrep.2022.110329)
Supplement: 1 [file NIHMS1776715-supplement-1.pdf]

Cell Reports, Volume *38*

Supplemental information

Gene silencing by EZH2 suppresses TGF-β activity within the decidua to avert pregnancy-adverse wound healing at the maternal-fetal interface

Ivan Osokine, Johan Siewiera, Damon Rideaux, Stephany Ma, Tatsuya Tsukui, and Adrian Erlebacher

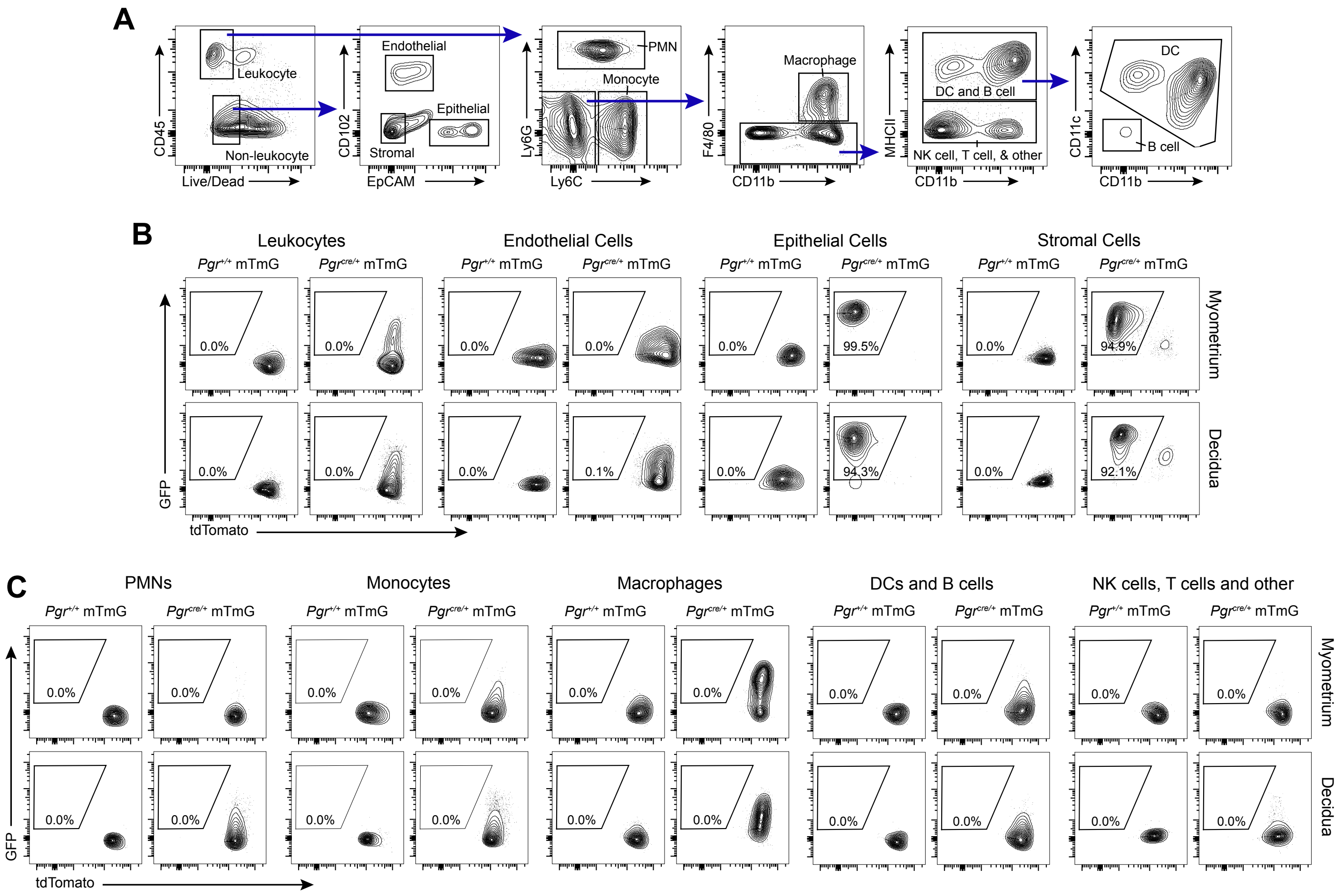

Supplemental Figure 1. *Pgr*-cre-mediated recombination is evident in the large majority of uterine stromal and epithelial cells, but not in uterine endothelial cells nor leukocytes. Related to Figure 1.

To determine which cell types within implantation sites of early pregnancy undergo *Pgr*-cre-mediated recombination, we employed a lineage tracing approach using mTmG mice (Muzumdar et al., 2007). These mice bear a ubiquitously and constitutively active tdTomato/GFP reporter construct that directs cells to express tdTomato prior to cre-mediated recombination and GFP post-recombination (with concomitant loss of tdTomato). Cells isolated from the myometrium and decidua of E7.5 implantation sites of *Pgr*$^{cre/+}$ mTmG mice and control *Pgr*$^{+/+}$ mTmG mice were analyzed by flow cytometry.

(A) Gating strategy. The decidua is shown but an identical strategy was applied to the myometrium. After identifying non-doublets based upon their forward- and side-scatter characteristics (not shown), cells were divided viable leukocyte (CD45^{+}) and non-leukocyte (CD45^{-}) populations. The non-leukocyte population was further subsetted into epithelial (EpCAM^{+}), endothelial (CD102^{+}) and stromal (EpCAM^{-} CD102^{-}) populations. Leukocytes were further divided into polymorphonuclear neutrophils (PMNs; Ly6G^{+} Ly6C^{int}), monocytes (Ly6G^{-} Ly6C^{+}), and a Ly6G/Ly6C double negative population that was in turn divided into F4/80^{+} macrophages, a F480^{-} MHCII^{+} population comprised of dendritic cells (DCs) and B cells, and a F480^{-} MHCII^{-} population presumably comprised mainly of natural killer (NK) cells but also expected to include T cells and other leukocytes otherwise not identified. The DCs (both CD11b^{lo} and CD11b^{hi} subsets; (Collins et al., 2009)) and B cells could be individually identified based on their expression of CD11c and CD11b, as shown, although we report recombination frequencies for the combined population due to the low numbers of B cells within the tissue.

(B) Recombination frequencies in stromal, epithelial, and endothelial cells, as well as total leukocytes. The gated GFP^{hi} tdTomato^{lo} population represents recombined cells. Note that the large majority of epithelial and stromal cells within the decidua and myometrium of *Pgr*$^{cre/+}$ mTmG mice have undergone *Pgr*-cre-mediated recombination. In contrast, endothelial cells and leukocytes in *Pgr*$^{cre/+}$ mTmG mice show a GFP$^{neg/lo}$ tdTomato^{hi} phenotype. This phenotype, with GFP expression particularly apparent in macrophages as per panel C, does not represent an early stage of GFP induction after a recent recombination event since the cells are all similarly GFP$^{neg/lo}$ tdTomato^{hi} on E18.5, 11 days later (data not shown). Thus, they do not appear to undergo recombination and their low GFP positivity likely represents their phagocytosis of GFP^{+} material derived from nearby stromal and epithelial cells. Data are representative of n = 5-8 mice/group.

(C) Recombination frequencies within leukocyte subpopulations. Data are representative of n = 3 *Pgr*$^{+/+}$ mTmG mice and n = 5 *Pgr*$^{cre/+}$ mTmG mice.

A

	Control	*Ezh2* cKO	*p*-value
Number of mice	6	6	
Total number of litters	9	4	
Litters per mouse (mean +/- SD)	1.5 +/- 0.5	0.7 +/- 0.5	p = 0.02
Litter size (mean +/- SD)	7.6 +/- 1.6	8.0 +/- 2.8	p = 0.72

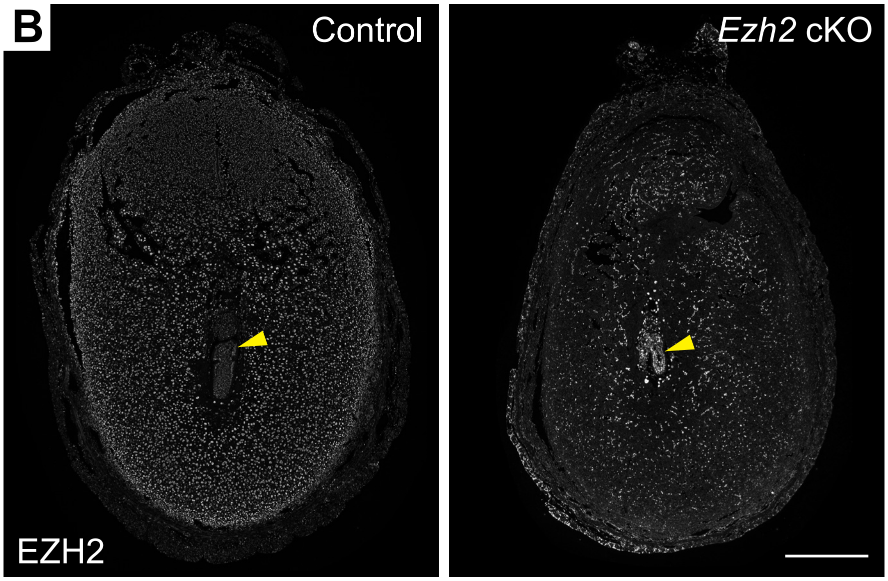

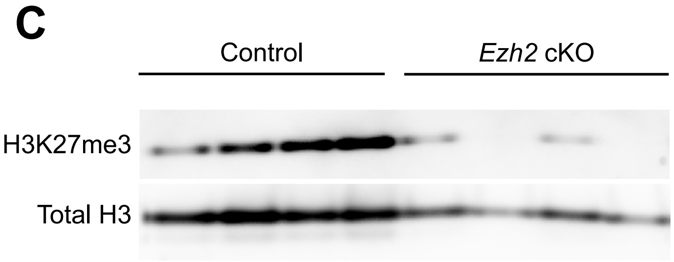

Supplemental Figure 2. Initial characterization of *Ezh2* cKO mice. Related to Figure 1.
(A) Fertility study. Control and *Ezh2* cKO mice, at 8-12 weeks of age, were housed with C57BL/6 males of proven fertility for a 10-week period. Live litter numbers and litter sizes were recorded.
(B) Immunofluorescence detection of EZH2 expression in control and *Ezh2* cKO E7.5 implantation sites. Although DSCs no longer express EZH2 in *Ezh2* cKO mice, endothelial cells and embryonic cells continue to stain EZH2-positive. Yellow arrowheads denote embryos. Representative images from n = 4 mice/group. Scale bar, 500 µm.
(C) Immunoblot for H3K27me3 and total H3 in whole tissue decidual lysates prepared from mice on E7.5. Data show one of two independent experiments each with n = 4 mice/group.

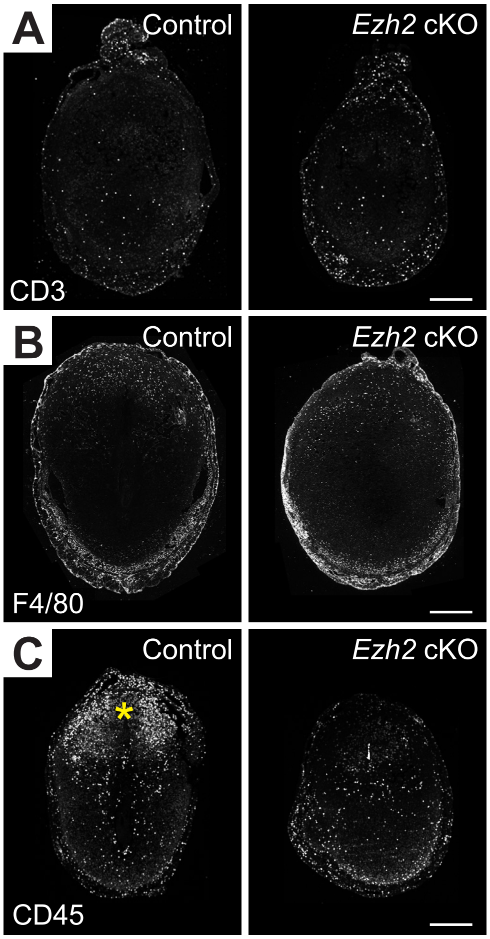

Supplemental Figure 3. ***Ezh2*** **cKO implantation sites do not accumulate leukocytes and instead show a paucity of uterine NK cells. Related to Figure 2.**
Immunofluorescence detection of (A) CD3, (B) F4/80, and (C) CD45 in E7.5 implantation sites, in order to visualize T cells, macrophages, and total leukocytes, respectively. Mice were injected daily with 2 mg P4 starting on E5.5. Asterisk indicates the characteristic accumulation of NK cells in the mesometrial pole of the control implantation site. Pixel intensities in panels A and C were dilated using the “maximum” filter of Image J software so that the rare positive cells would be visible in these panoramic images. Representative images from three independent experiments with n = 3-4 mice/group. Scale bars, 500 μm.

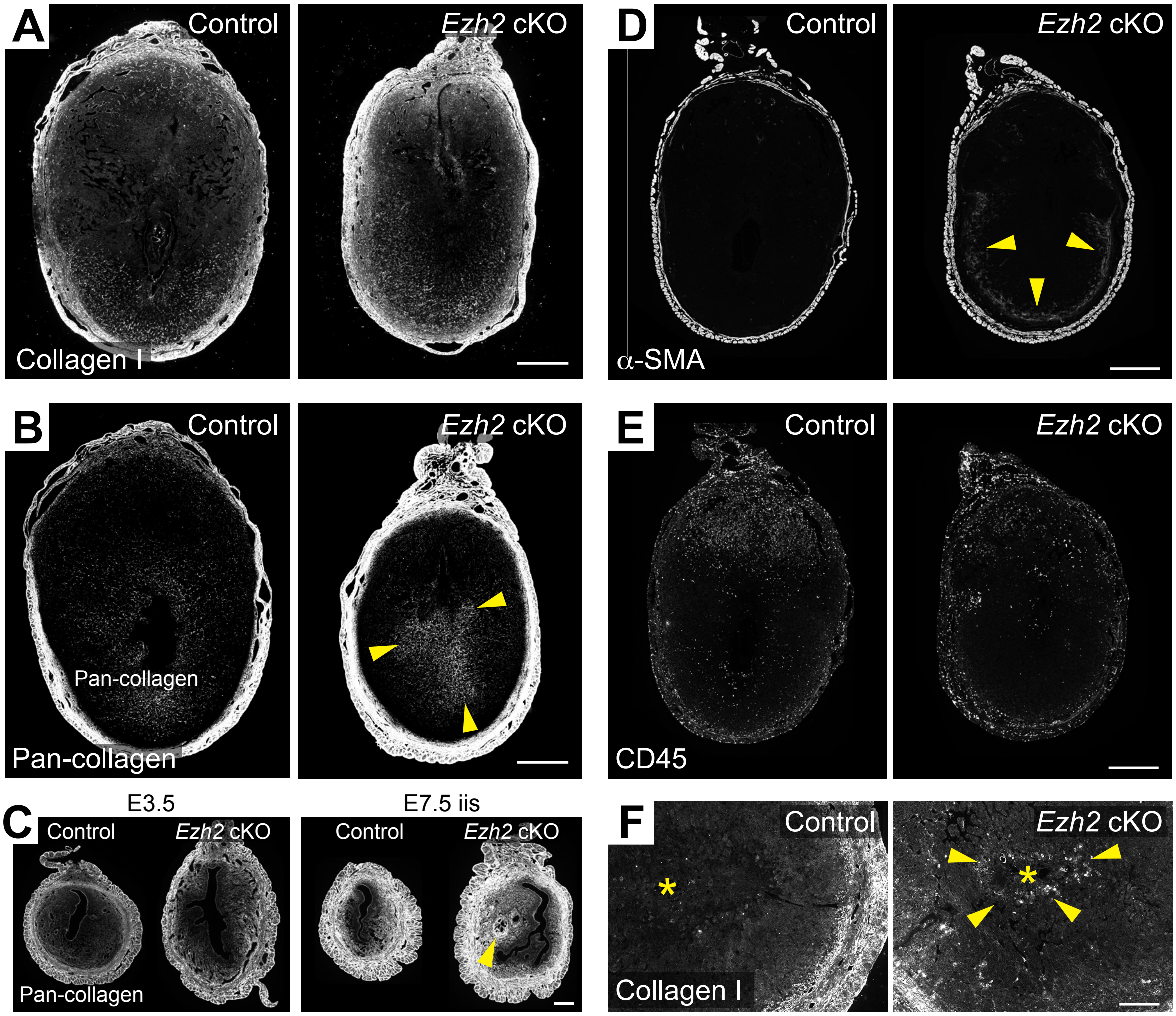

Supplemental Figure 4. Further analysis of peri-embryonic collagen deposition in the *Ezh2* cKO decidua; demonstration that α-SMA upregulation, NK cell paucity, and peri-embryonic collagen deposition are also evident in the decidua of *Ezh2* cKO mice not treated with P4. Related to Figure 2.

Mice sacrificed on E7.5 were either injected daily with 2 mg P4 starting at E5.5 (A-C) or left untreated (D-F).

(A and B) Detection of (A) type I collagen, and (B) total collagen within E7.5 implantation sites. Type I collagen was detected by immunofluorescence (representative images from $n = 5$ mice/group; close-ups of these same images are shown in Figure 2D); total collagen was detected using a pan-collagen detection reagent (Cy3-conjugated Collagen Hybridizing Peptide from 3Helix) (representative images from $n = 4$ mice/group; arrowheads indicate peri-embryonic collagen accumulations in *Ezh2* cKO implantation sites). Scale bars, 500 µm.

(C) Expression of total collagen in undecidualized control and *Ezh2* cKO uteri on E3.5 or within E7.5 inter-implantation sites. Arrowhead indicates peri-glandular collagen deposition. Note also that these uteri, which are from 8-12 week old mice, show no evidence of epithelial hyperplasia. Representative images from $n = 3$ mice/group. Scale bar, 200 µm.

(D-F) Immunofluorescence detection of (D) α-SMA, (E) CD45, or (F) type I collagen expression in representative sections of E7.5 implantation sites from control and *Ezh2* cKO mice not treated with P4. The ring of α-SMA^+ cells in the antimesometrial decidua (arrowheads, D) was observed in $n = 21/21$ *Ezh2* cKO and 2/25 control implantation sites from $n = 4$ mice/group ($P < 0.0001$, Fisher's exact test). Peri-embryonic collagen accumulations (arrowheads, F) were observed in $n = 5/5$ *Ezh2* cKO decidua and $n = 1/6$ control decidua from $n = 4$ mice/group ($P = 0.015$, Fisher's exact test). Asterisks indicate embryos. Note the high density of type I collagen staining in the surrounding myometrium. Scale bars: (D and E), 500 µm; (F), 200 µm.

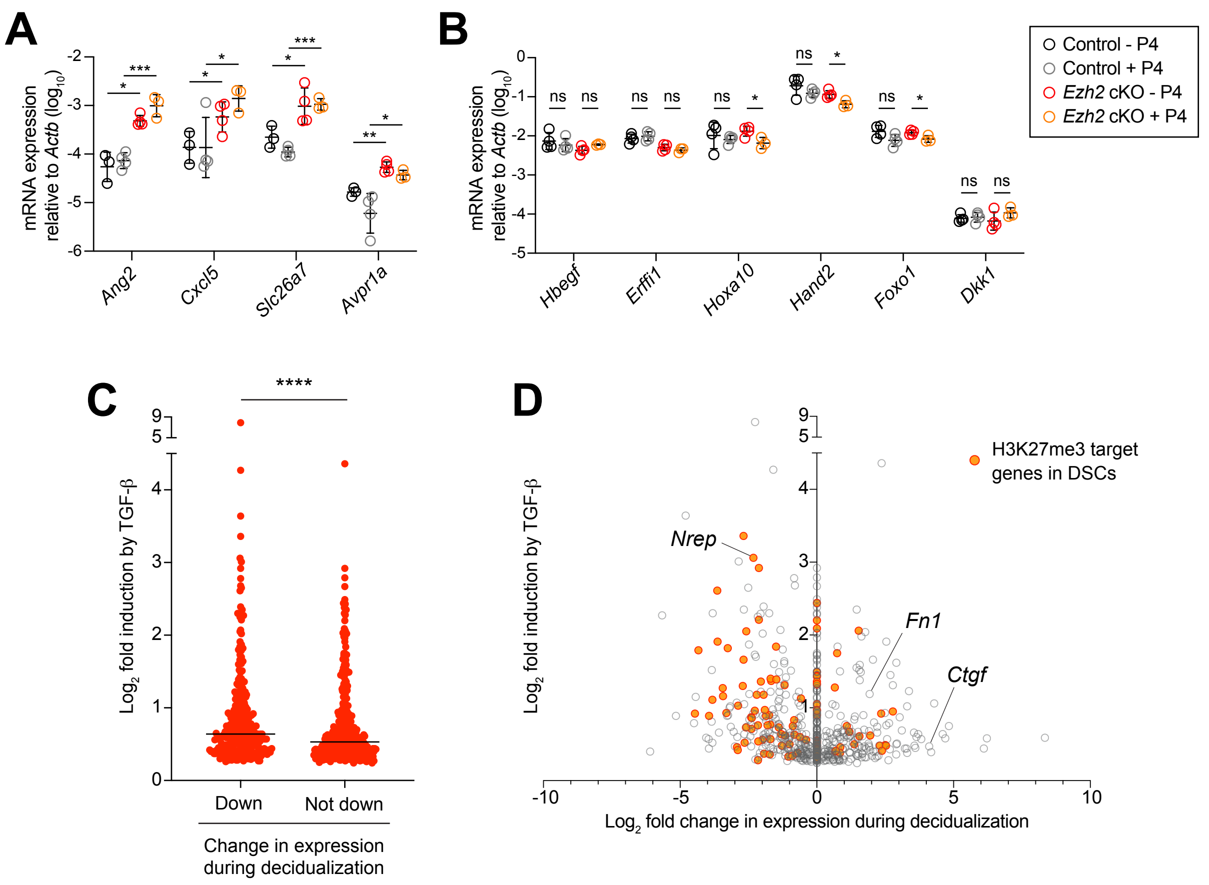

Supplemental Figure 5. Additional analysis of gene expression in the *Ezh2* cKO decidua. Related to Figures 3 and 5.
(A and B) qRT-PCR analysis of gene expression. RNA was extracted from whole decidual tissue from E7.5 implantation sites of control and *Ezh2* cKO mice that were either untreated (-P4) or injected daily with 2 mg P4 starting on E5.5 (+P4). Panel A shows genes that were amongst the top 10 most upregulated in the *Ezh2* cKO decidua as revealed by our RNA-seq analysis of intact tissues (Supplemental Table 1). The specific genes analyzed were chosen based upon whether we could design good primer sets common to all splice variants. Note that increased expression of these genes in the *Ezh2* cKO decidua was similarly apparent whether or not the mice received P4. Indeed, with serum levels of endogenous P4 already dramatically elevated in early gestation, P4 supplementation appeared to have no major effect on decidual physiology since a set of putative P4 target genes (Wetendorf and DeMayo, 2012) showed no differences in expression between P4-treated and -untreated control mice (B). For *Ezh2* cKO mice, statistically significant expressions differences in these genes were minor and did not cause absolute expression levels to fall outside the range displayed by control mice. Data show geometric mean ± geometric SD, $n = 3$-4 mice/group; *, $P < 0.05$; **, $P < 0.01$; ***, $P < 0.001$ by Student's *t*-test.
(C and D) Comparisons between the fold induction of TGF-β target genes in *Ezh2* cKO E7.5 DSCs following TGF-β treatment and the respective change in these genes' expression that occurs with decidualization in wild-type mice (using the same parameters as the analysis of control DSCs in Figures 5A and 5B). For panel (C), we grouped the genes according to whether they were downregulated during decidualization (i.e., they showed significantly [*FDR* < 0.05] lower expression in DSCs compared to USCs; $n = 264$), or not (i.e., they showed significantly higher expression or no significant change in expression compared to USCs; $n = 355$). Lines denote median TGF-β inducibility. ****, $P = 0.0002$, Mann-Whitney test. For panel (D), we plotted fold induction in explicit relationship to the fold change in expression between DSCs and USCs, with genes showing no significant difference between DSCs and USCs plotted at 0.0.

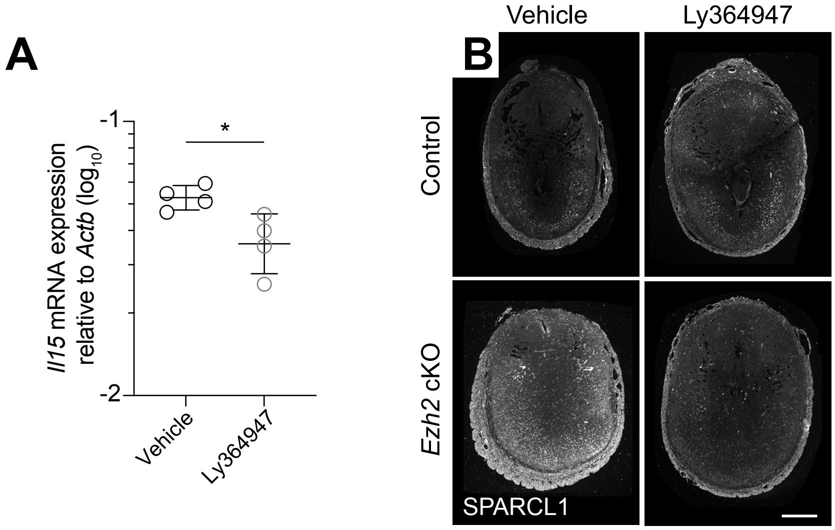

Supplemental Figure 6. **Ly364947 treatment reduces *Il15* expression in the wild-type decidua and SPARCL1 expression in the *Ezh2* cKO decidua. Related to Figure 6.**
Mice were injected daily with 2 mg P4 and either vehicle (20% DMSO) or Ly364947 starting on E5.5.
(A) qRT-PCR analysis of decidual *Il15* mRNA expression in C57BL/6 mice on E8.5. Data show geometric mean ± geometric SD, $n = 4$ mice/group; *, $P < 0.05$ by Student's t-test.
(B) Immunofluorescence detection of the TGF-β target SPARCL1 in E7.5 implantation sites of control and *Ezh2* cKO mice. Of note, despite the increase in SPARCL1 protein levels in the vehicle-treated *Ezh2* cKO decidua, *Sparcl1* mRNA levels were not correspondingly elevated (Supplemental Table 1) suggesting that *Sparcl1* expression is only transiently induced upon implantation. Representative images from $n = 5$ mice/group; scale bar, 500 μm.

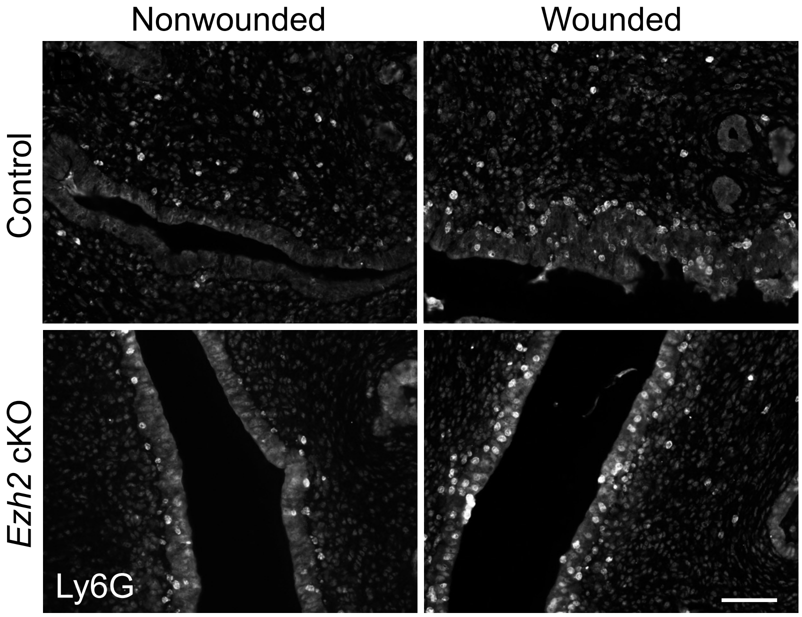

Supplemental Figure 7. Neutrophils accumulate in the non-pregnant control and *Ezh2* cKO uterus after wounding. Related to Figure 7.
Immunofluorescence detection of Ly6G$^+$ neutrophils in the uteri of nonwounded or wounded control or *Ezh2* cKO mice. Note the sub- and intra-epithelial accumulation of neutrophils in wounded uteri. For each mouse (*n* = 3 per group), one uterine horn was pierced at multiple locations with a fine needle, without lentiviral injection; the contralateral uterine horn was unmanipulated to serve as an internal control. Scale bar, 50 μm.

Supplemental Table 5. Antibodies and experimental conditions for immunofluorescence. Related to Figures 2, 6, 7 and Supplemental Figures 2, 3, 4, 6, 7.

Target antigen	Species	Clone, if monoclonal	Source	Catalogue Number	Retrieval	Dilution	Amplification
Alpha-smooth muscle actin	rat	1A4	eBioscience	53-9760-82	Any	1:100	No
CD3	rabbit	polyclonal	Agilent	A045229-2	Any	1:1000	Yes
CD45	rabbit	polyclonal	Abcam	ab10558	TE	1:4000	Yes
E2F8	rabbit	polyclonal	Signalway Antibody	32173	TE	1:1000	Yes
EZH2	rabbit	SP129	Acris Origene	AM33207PU-S	TE	1:1000	Yes
F4/80	rat	C1:A3-1	Cedarlane	CL8940AP	Trypsin	1:200	Yes
GFP	rabbit	polyclonal	Novus Biologicals	NB600-308	TE	1:1000	Yes
LY6G	rat	1A8	BD Pharmingen	551459	Citrate	1:250	No
SPARCL1	goat	polyclonal	R&D Systems	AF2836	TE	1:1000	Yes
Type I collagen	rabbit	polyclonal	Abcam	ab34710	Trypsin	1:500	Yes

Supplemental Table 6. Primers for qRT-PCR. Related to Supplemental Figures 5 and 6.

**Gene:**	**Forward (5'->3'):**	**Reverse (5'->3"):**
Actb	GCTCTGGCTCCTAGCACCAT	GCCACCGATCCACACAGAGT
Hbegf	TGCAAATGCCTCCCTGGTTA	TCCTCTCCTGTGGTACCTAAAC
Erffi1	TGATCCAATAACCATGGCCTACAA	GACCGAGCAGCACATCCAAC
Hoxa10	AGAGCAGCACGGTACGTTAT	TCGCCTTTGGAACTGCCTTG
Hand2	CCAGCTACATCGCCTACCTC	GTGCTTTTCAAGATCTCATTCAGC
Foxo1	AACCAAAGCTTCCCACACAGT	AAGGACTTTTAAATGTAGCCTGC
Dkk1	GACAACTACCAGCCCTACCC	GATCTGTACACCTCCGACGC
Ang2	ATGAGCCCAGGTCCTTTGTT	AGAGGTTAGCTTTCTTTTCACCAT
Cxcl5	TTCAGAAAATATTGGGCAGTGAC	GAAAATCCGTGGGTGGAGAGA
Il15	CATTTTGGGCTGTGTCAGTGT	TGCAGTAACTTTGCAACTGGG
Slc26a7	ACGGATTAGAAGTAGTTGGGCA	TGGGCCAAAAATTCCTGGTTG
Avpr1a	TTCGTTTGGACCGATTCCGA	GTCTCCGGCTCATGCTATCC
